# Association of bronchial steroid inducible methylation quantitative trait loci with asthma and chronic obstructive pulmonary disease treatment response

**DOI:** 10.1002/clt2.12173

**Published:** 2022-08-29

**Authors:** Elise M. A. Slob, Alen Faiz, Jos van Nijnatten, Susanne J. H. Vijverberg, Cristina Longo, Merve Kutlu, Fook Tim Chew, Yang Yie Sio, Esther Herrera‐Luis, Antonio Espuela‐Ortiz, Javier Perez‐Garcia, Maria Pino‐Yanes, Esteban G. Burchard, Uroš Potočnik, Mario Gorenjak, Colin Palmer, Cyrielle Maroteau, Steve Turner, Katia Verhamme, Leila Karimi, Somnath Mukhopadhyay, Wim Timens, Pieter S. Hiemstra, Mariëlle W. Pijnenburg, Margaret Neighbors, Michele A. Grimbaldeston, Gaik W. Tew, Corry A. Brandsma, Vojko Berce, Hananeh Aliee, Fabian Theis, Don D. Sin, Xuan Li, Maarten van den Berge, Anke H. Maitland‐van der Zee, Gerard H. Koppelman

**Affiliations:** ^1^ Department of Respiratory Medicine, Amsterdam University Medical Centers University of Amsterdam Amsterdam The Netherlands; ^2^ Department of Paediatric Pulmonology, Amsterdam Public Health Research Institute, Amsterdam University Medical Centers University of Amsterdam Amsterdam The Netherlands; ^3^ Department of Clinical Pharmacy Haaglanden Medical Center The Hague The Netherlands; ^4^ Department of Pulmonology University Medical Center Groningen Groningen The Netherlands; ^5^ University of Groningen, University Medical Center Groningen, Groningen Research Institute for Asthma and COPD Groningen The Netherlands; ^6^ Respiratory Bioinformatics and Molecular Biology, School of Life Sciences University of Technology Sydney Sydney New South Wales Australia; ^7^ Division of Pharmacoepidemiology and Clinical Pharmacology Utrecht Institute for Pharmaceutical Sciences Faculty of Science Utrecht University Utrecht The Netherlands; ^8^ Department of Biological Science National University of Singapore Singapore Singapore; ^9^ Genomics and Health Group, Department of Biochemistry, Microbiology, Cell Biology and Genetics Universidad de La Laguna Santa Cruz de Tenerife Spain; ^10^ CIBER de Enfermedades Respiratorias Instituto de Salud Carlos III Madrid Spain; ^11^ Instituto de Tecnologías Biomédicas (ITB) Universidad de La Laguna San Cristóbal de La Laguna Santa Cruz de Tenerife Spain; ^12^ Department of Medicine University of California San Francisco San Francisco California USA; ^13^ Department of Bioengineering and Therapeutic Sciences University of California San Francisco San Francisco California USA; ^14^ Center for Human Molecular Genetics and Pharmacogenomics, Faculty of Medicine University of Maribor Maribor Slovenia; ^15^ Laboratory for Biochemistry, Molecular Biology and Genomics, Faculty for Chemistry and Chemical Engineering University of Maribor Maribor Slovenia; ^16^ Population Pharmacogenetics Group, Biomedical Research Institute University of Dundee Dundee UK; ^17^ Child Health University of Aberdeen Aberdeen UK; ^18^ Department of Medical Informatics Erasmus Medical Center Rotterdam The Netherlands; ^19^ Department of Paediatrics Royal Alexandra Children's Hospital Brighton UK; ^20^ Department of Pathology and Medical Biology University of Groningen, University Medical Center Groningen Groningen The Netherlands; ^21^ Department of Pulmonology Leiden University Medical Center Leiden The Netherlands; ^22^ Department of Pediatrics, Pediatric Pulmonology and Allergology Erasmus Medical Center‐Sophia Children's Hospital Rotterdam The Netherlands; ^23^ OMNI Biomarker Development, Genentech Inc South San Francisco California USA; ^24^ Product Development Immunology, Infectious Disease & Ophtalmology Genentech Inc South San Francisco California USA; ^25^ Department of Pediatrics University Medical Centre Maribor Maribor Slovenia; ^26^ Institute of Computational Biology, Helmholtz Center Munich Germany; ^27^ Department of Mathematics Technical University of Munich Munich Germany; ^28^ Centre for Heart Lung Innovation St. Paul's Hospital and University of British Columbia Vancouver British Columbia Canada; ^29^ Department of Pediatric Pulmonology and Pediatric Allergology University of Groningen, University Medical Center Groningen, Beatrix Children's Hospital Groningen The Netherlands

**Keywords:** children, exacerbations, inhaled corticosteroids, methylation quantitative trait loci (meQTL), pharmacogenetics

To the editor,

Large variation in response to inhaled corticosteroids (ICS) has been reported in both asthma and chronic obstructive pulmonary disease (COPD), which may partly be explained by genetic factors. The transcriptome of the airways changes following ICS treatment,^1^ which may be directed by single nucleotide polymorphisms (SNPs), that affect deoxyribonucleic acid (DNA) methylation (methylation‐Quantitative Trait Loci, meQTL).

A strong and consistent response of the airways to ICS in both asthma and COPD patients^1^, ^2^ has been found, and severe childhood asthma has been associated with increased odds of COPD development in later life,^3^ showing that overlap between the diseases may exist. We hypothesised that preselection of steroid‐inducible meQTL that affect DNA methylation upon ICS treatment may increase power to find SNPs that also clinically affect response to ICS and that these genetic variants might overlap between asthma and COPD. The aim of this study was to identify SNPs that affect change in DNA methylation in the airway wall upon ICS treatment, and to investigate whether these SNPs are associated with asthma exacerbations in children despite treatment with ICS.

For the identification of meQTLs, we investigated 43 Dutch COPD patients from the Groningen and Leiden Universities study of Corticosteroids in Obstructive Lung Disease (GLUCOLD) study (Table S1).^1^ Longitudinal airway wall DNA methylation (EPIC 850 K array) and gene expression (ribonucleic acid‐sequencing, RNA‐seq) was collected from these patients pre‐ and post‐6 months of fluticasone ± salmeterol (500/50 μg twice daily) treatment (Figure S1). We focused on methylation sites that previously were shown to be altered during ICS treatment (1049 CpG sites).^4^ This analysis identified 76 inducible meQTL caused by 71 independent SNPs with an false discovery rate (FDR) < 0.05 (Table S2). The most significant association was between cg13086983 and rs10917023, where the G allele (minor allele frequency: 7.7%) induced higher methylation (Beta: 0.849, *p* value: 4.21 × 10^−06^). Of these 76 CpG sites, 24 were associated with 24 gene transcripts (Table S3). The most significant association was found between the Cytosine‐phosphate‐Guanine (CpG) site cg08570199 and the *CCDC80* gene (Beta coefficient: −1.249, *p*‐value: 2.05 × 10^−4^; Figure 1A–D).

**FIGURE 1:**
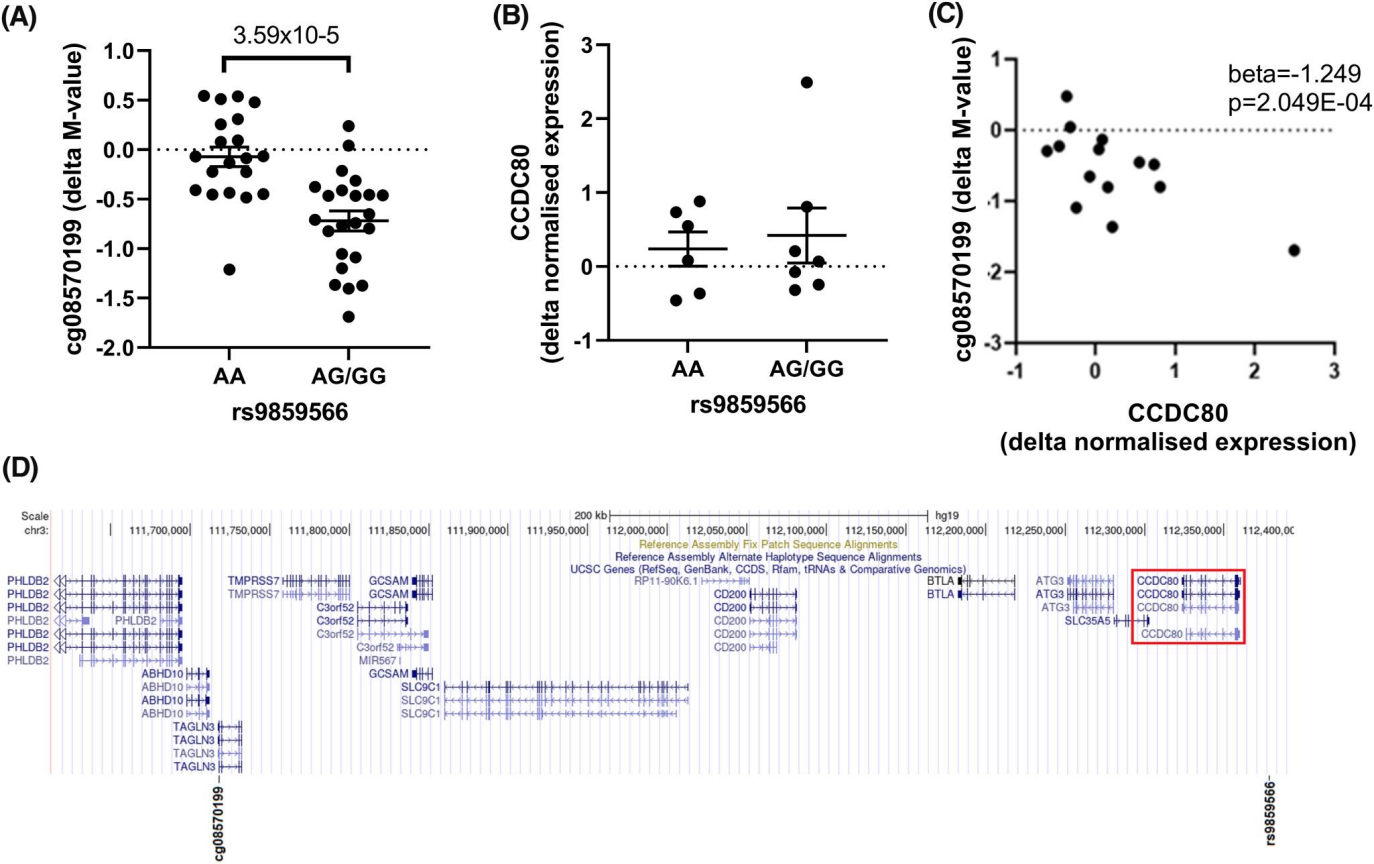
The influence of genetics on the changes of methylation during corticosteroid treatment. (A) Inducible meQTL analysis of the change in cg08570199 methylation before and 6 months following ICS treatment in bronchial biopsies and the SNP rs9859566. (B) Change in CCDC80 gene expression before and 6 months following ICS treatment in bronchial biopsies separated by the genotype of rs9859566. (C) EQTM analysis of the change in cg08570199 methylation and CCDC80 gene expression following before and 6 months following ICS treatment in bronchial biopsies. (D) Diagram of the relative positions of rs9859566 and cg08570199 to the gene CCDC80

Subsequently, we investigated whether the identified meQTL were associated with asthma or COPD exacerbations despite ICS use in children with asthma and adult COPD patients, respectively. The asthma analysis was conducted performing a meta‐analysis in eight cohort studies from the Pharmacogenomics in Childhood Asthma (PiCA) consortium^5^ stratified by European (*n* = 1515) or non‐European descent (*n* = 1702) (see online supplementary Table and S4). Two outcomes were defined according to the American Thoracic Society/European Respiratory Society 2009 statement: (1) ‘any exacerbation’: hospitalisations, asthma‐related emergency room visits, or oral corticosteroids (OCS) courses in the past 6–12 months and (2) OCS courses in the past 6–12 months. None of the identified meQTL were associated with the outcomes ‘any’ exacerbations and OCS courses in the past 6–12 months (Tables S5–S8). The COPD analysis was conducted in the Lung Health Study (LHS)‐2, including 1116 COPD patients (ICS, *n* = 559 or placebo, *n* = 557) with lung function measurements over 3 years,^6^ where a previous pharmacogenomic analysis focusing on genotype‐by‐ICS treatment effect on 3 years of FEV1 changes (estimated as slope) was investigated for the 71 SNPs None of the identified meQTL SNPs were associated with FEV1 decline after multiple testing correction (Table S9).

In conclusion, corticosteroid inducible meQTL analysis enabled us to identify a set of functional SNPs that may be useful for future (functional) studies. Although our results indicate that overlap in genetic response to steroids may exist, comparing the epigenetic responses in adult COPD patients to clinical effects in children with asthma, we do acknowledge that additional, disease and age‐specific effects may be present. However, these SNPs were not significantly associated with exacerbations and OCS courses in children nor with the slope of FEV1 in adults with COPD.

## AUTHOR CONTRIBUTIONS

Elise M. A. Slob contributed to the statistical analysis and interpretation of the data, design of tables and figures, writing of the original draft and review and editing. Alen Faiz contributed to the conception and design, statistical analysis and interpretation of data, design of tables and figures, writing of the original draft and review and editing. Jos van Nijnatten contributed to the statistical analysis and design of the tables and figures and review and editing. Susanne J. H. Vijverberg contributed to the subject recruitment, data collection and review and editing. Maria Pino‐Yanes contributed to the subject recruitment, data collection and review and editing. Esteban G. Burchard contributed to the subject recruitment, data collection and review and editing. Uroš Potočnik contributed to the subject recruitment, data collection and review and editing. Colin Palmer contributed to the subject recruitment, data collection and review and editing. Steve Turner contributed to the subject recruitment, data collection and review and editing. Katia Verhamme contributed to the subject recruitment, data collection and review and editing. Somnath Mukhopadhyay contributed to the subject recruitment, data collection and review and editing. Leila Karimi contributed to the subject recruitment, statistical analysis and interpretation of the data, data collection and review and editing. Fook Tim Chew contributed to the subject recruitment, data collection and review and editing. Wim Timens contributed to the subject recruitment, data collection and review and editing. PH contributed to the subject recruitment, data collection and review and editing. Mariëlle W. Pijnenburg contributed to the interpretation of the data and review and editing. Maarten van den Berge contributed to the subject recruitment, conception and design, data collection and review and editing. Hananeh Aliee contributed to the subject recruitment, data collection and review and editing. Vojko Berce contributed to the subject recruitment, data collection and review and editing. Corry A. Brandsma contributed to the subject recruitment, data collection and review and editing. Margaret Neighbors contributed to the subject recruitment, data collection and review and editing. Michele A. Grimbaldeston contributed to the subject recruitment, statistical analysis and data interpretation, data collection and review and editing. Gaik W. Tew contributed to the subject recruitment, data collection and review and editing. Antonio Espuela‐Ortiz contributed to the statistical analysis and interpretation of the data and review and editing. Yang Yie Sio contributed to the statistical analysis and interpretation of the data and review and editing. Javier Perez‐Garcia contributed to the statistical analysis and interpretation of the data and review and editing. Merve Kutlu contributed to the statistical analysis and interpretation of the data and review and editing. Don D. Sin contributed to the subject recruitment, data collection and review and editing. Xuan Li contributed to the subject recruitment, data collection and review and editing. Anke H. Maitland contributed to the subject recruitment, data collection and review and editing. Cyrielle Maroteau contributed to the statistical analysis and interpretation of the data and review and editing. Gerard H. Koppelman contributed to the conception and design, statistical analysis and interpretation of data, writing of the original draft, review and editing and is the principle investigator. All authors approved the final version of the manuscript.

## CONFLICT OF INTEREST

E.M.A. Slob, A. Faiz, S.J.H. Vijverberg, H. Aliee, J. van Nijnatten, P. Hiemstra, C. Maroteau, C. Palmer, M. Kutlu, S. Turner, S. Mukhopadhyay, M. Gorenjak, C.A. Brandsma, L. Karimi, Y.Y. Sio, C. Longo and M.W. Pijnenburg have nothing to disclose. F.T. Chew reports grants from Singapore Ministry of Education Academic Research Fund, Singapore Immunology Network, National Medical Research Council (NMRC) (Singapore), Biomedical Research Council (BMRC) (Singapore), and the Agency for Science Technology and Research (A*STAR) (Singapore), during the conduct of the study; and consulting fees from Sime Darby Technology Centre; First Resources Ltd; Genting Plantation, and Olam International, outside the submitted work. M. Pino‐Yanes reports grants from the Spanish Ministry of Science, Innovation, and Universities, the State Research Agency, and the European Regional Development Fund from the European Union (MICIU/AEI/FEDER, UE). E. Herrera‐Luis and J. Perez‐Garcia report a fellowships from MICIU. A. Espuela‐Ortiz declares a fellowship granted by MICIU/ULL. E.G. Burchard reports grants from the National Institutes of Health, the Tobacco‐Related Disease Research Program, the Sandler Family Foundation, the American Asthma Foundation, the Amos Medical Faculty Development Program from the Robert Wood Johnson Foundation, and from the Harry Wm. and Diana V. Hind Distinguished Professorship in Pharmaceutical Sciences II. W. Timens reports fees to the UMCG from Roche Diagnostics/Ventana, personal fees from Merck Sharp Dohme, Bristol‐Myers‐Squibb and AbbVie, outside the submitted work. G.H. Koppelman reports grants from Lung Foundation of the Netherlands, UBBO EMMIUS Foundation, TETRI Foundation, TEVA the Netherlands, Vertex, GSK, European Union (H2020 Prominent Grant), outside the submitted work; and he has acted on advisory board meetings of GSK and Pure IMS, outside the submitted work. A.H. Maitland‐van der Zee has received research grants outside the submitted work from GSK, Boehringer Íngelheim and Vertex, she is the PI of a P4O2 (Precision Medicine for more Oxygen) public private partnership sponsored by Health Holland involving many private partners that contribute in cash and/or in kind (Boehringer Ingelheim, Breathomix, Fluidda, Ortec Logiqcare, Philips, Quantib‐U, Smartfish, SODAQ, Thirona, TopMD and Novartis), and she has served in advisory boards for AstraZeneca, GSK and Boehringer Ingelheim with money paid to her institution. Dr. van den Berge reports grants to University from GlaxoSmithKline, Novartis, Genentech, outside the submitted work. Dr. Potočnik reports grants from Slovenian Research Agency, grants from Ministry of Education, Science and Sport Slovenia (MIZS), during the conduct of the study. M. Grimbaldeston, G.W. Tew and M. Neighbors are employees of Genentech Inc., a Member of the Roche Group. K. Verhamme works for a research department which receives/received unconditional research grants from Yamanouchi, Pfizer‐Boehringer Ingelheim, Novartis, GSK, Chieisi, Amgen, UCB, none of which are related to the content of this work. F.J. Theis acknowledges support by the BMBF (grant #L031L0214A, grant# 01IS18036B and grant# 01IS18053A) and by the Helmholtz Association's Initiative and Networking Fund through Helmholtz AI [grant number: ZT‐I‐PF‐5‐01] and sparse2big [grant number ZT‐I‐007]. V. Berce reports grants from Slovenian Research Agency, grants from Ministry of Education, Science and Sport Slovenia (MIZS), during the conduct of the study.

## FUNDING INFORMATION

The Netherlands Organisation for Scientific Research; the Dutch Asthma Foundation; GlaxoSmithKline, the University Medical Center Groningen; Leiden University Medical Center. Genentech Inc.; Lung Foundation Netherlands, Grant/Award Number: number 5.1.16.094; GlaxoSmithKline and Utrecht Institute for Pharmaceutical Sciences; Slovenian Research Agency, Grant/Award Number: P3‐0067; SysPharmPedia grant; the Ministry of Education, Science and Sport Slovenia (MIZS), Grant/Award Number: C3330‐16‐500106; Sandler Family Foundation; The American Asthma Foundation, the RWJF Amos Medical Faculty Development Program; The National Heart, Lung, and Blood Institute of the National Institutes of Health, Grant/Award Number: R01HL117004, R01HL128439, R01HL135156, X01HL134589, R01HL141992, R01HL141845; National Institute of Health and Environmental Health Sciences, Grant/Award Number: R01ES015794, R21ES24844; The National Institute on Minority Health and Health Disparities, Grant/Award Number: P60MD006902, RL5GM118984, R01MD010443, and R56MD013312; the Tobacco‐Related Disease Research Program, Grant/Award Number: 24RT‐0025, 27IR‐0030; National Human Genome Research Institute, Grant/Award Number: U01HG009080; ISCIII and ERDF; Ramón y Cajal Program by the Spanish Ministry of Science, Innovation, and Universities, Grant/Award Number: RYC‐2015‐17205; MICIU, FPU19/02175; The State Research Agency; The European Union, Grant/Award Number: MICIU/AEI/FEDER, UE, SAF2017‐83417R; MICIU fellowship, Grant/Award Number: PRE2018‐083837; MICIU/ULL (M‐ULL); Slovenian Research Agency, Grant/Award Number: P3‐0067; SysPharmPedia, The Ministry of Education, Science and Sport Slovenia, Award Number: C3330‐16‐500106; Roche Diagnostics GmbH and Cellarity Inc.

## Supporting information

Supporting Information S1

## Data Availability

Research data are not shared.
