# Supplementary material for: Association of bronchial steroid inducible methylation quantitative trait loci with asthma and chronic obstructive pulmonary disease treatment response
Source: Clin Transl Allergy. 2022 Aug 29;12(8):e12173. doi: 10.1002/clt2.12173 (PMC9421427; doi:10.1002/clt2.12173)
Supplement: Supplementary file 1 — Supporting Information S1 [file CLT2-12-e12173-s001.docx]

**Supplementary information - Methods**

*Steroid inducible meQTL analysis:* We included a subset of 43 COPD patients from the Groningen and Leiden Universities study of Corticosteroids in Obstructive Lung Disease (GLUCOLD), of whom genome-wide genotype information obtained by means of the *Illumina HymanCytoSNP-12* array was available. The bronchial biopsy collection has been previously explained in detail[1]. Longitudinal airway wall gene expression (RNA-seq) and DNA methylation was collected from these patients pre- and post-six months of fluticasone ± salmeterol (500/50 µg twice daily) treatment, to identify inducible meQTL (Supplementary Figure 1). We focused on methylation sites that have previously been shown to be altered during ICS treatment (1,049 CpG sites)[2]. Methylation profiling was conducted using Infinium MethylationEPIC 850K, further information is described in Supplementary information 1. RNA-seq has been previously described[3].

*GLUCOLD study:* A Dutch COPD cohort of patients with no history of of asthma. The inclusion criteria for the GLUCOLD study has been previously described (NCT00158847)[4]. In the GLUCOLD study, patients were randomly assigned to receive one of four treatments in a blinded way for patients, clinicians and researchers: (1) fluticasone 500 μg twice daily for 30 months; (2) fluticasone/salmeterol 500/50 μg twice daily for 30 months, (3) placebo twice daily for 30 months or (4) fluticasone 500 μg twice daily for the first 6 months followed by placebo twice daily for 24 months. Arms 1, 2 and 4 for selected for the analysis and samples were compared between baseline and 6 months treatment. The study was approved by the local medical ethics committees and all patients gave their written informed consent.

*Association of meQTL with clinical characteristics: candidate-gene studies.* A candidate-gene meta-analysis was carried out including eight cohort studies from the Pharmacogenomics in Childhood Asthma (PiCA) consortium: BREATHE, PAGES, PACMAN, SCSGES, Slovenia, GALA II, SAGE, and ESTATe for analysis. All participating studies have genome-wide data available from children with asthma treated with ICS and short-acting beta2-agonists (SABA), as well as data describing asthma exacerbations, as described elsewhere[5]. The designs and genotyping methods of the eight included cohort studies are summarised in Supplementary Information 2. All genomic datasets performed imputation and quality control (Hardy Weinberg Equilibrium cut-off 0.001, Minor Allele Frequency cut-off 1%, call rate per SNP > 95% and per individual >= 95%). The SNPs that were available after quality control procedures were included in the analyses. All children and their parents provided written informed consent and the studies were approved by their local ethics committees. Lung Health Study (LHS)-2: contained 1116 COPD patients which were treated with either ICS triamcinolone acetonide (n=559) or placebo (n=557) with spirometry performed every 6 months for 3 years. Further details are provided in the following publication [6].

*Outcomes of the candidate-gene studies:*

The presence of information on exacerbations was assessed for each study. A severe exacerbation was considered as a short course (3–5 days) of OCS use or a hospitalization/emergency room (ER) visit according to the American Thoracic Society/European Respiratory Society (ATS/ERS) 2009 statement[7].

Oral steroids were used as a “stand in” for exacerbations. In some cases patients were not admitted to the hospital or the emergency room, but seen by a GP and were given oral corticosteroids to treat the exacerbation. Because this may be a different group of patients with a less severe exacerbation, we also performed the analysis separately.

In the COPD study, 3 years of FEV_1_ changes (estimated as slope) during treatment was compared between placebo and ICS treatment.

**Statistical analysis**

For the inducible meQTL analyses in COPD patients, linear models were used to assess the association of SNPs (dominant model) with the delta DNA methylation prior and post six months ICS treatment, with correction for age, gender, smoking status, PC1 and PC2 as these factors are well known to influence DNA methylation. The window size used for this analysis was 1 Mb flanking the start site of the measured gene. The inducible meQTL analyses were performed using the R package MatrixEQTL version 2.2. After identification of inducible meQTL, we determined whether these CpGs were associated with changes in gene expression during ICS treatment through an inducible expression quantitative trait methylation (eQTM) analysis, for samples that had both DNA methylation and gene expression data at both time points via linear models including PC1 and PC2 using the R package MatrixEQTL version 2.2. The R package limma was used to normalise gene expression and methylation as response variable in the eQTM analysis[8]. A false discovery rate (FDR) of 5% was used to declare statistical significance. Only the meQTLs associated with eQTMs were carried forward into the candidate-gene analysis. To perform the candidate-gene analysis, generalised logistic regression models were used to assess the association between the 57 available SNPs after imputation and quality control derived from the meQTL analysis and asthma exacerbations despite ICS use using an additive model adjusting for age, gender and genetic principal components in the studies including admixed individuals. Analyses were stratified by European descent or non-European descent. Random effects models were used in the meta-analysis considering the heterogeneity in the included studies. Significance threshold was adjusted by means of Bonferroni correction resulting in a significant p-value threshold of 8.8 x 10^-4^ (0.05/57) for meQTL association. SNP extraction was performed with PLINK v1.90. To investigate robustness of findings, we performed a sensitivity analysis where we performed the same analysis for the significant SNPs from the candidate-gene analysis in SABA only users. Statistical analyses were carried out using IBM SPSS version 25.0 for Windows (SPSS Inc., Chicago, IL, USA) and R version 3.6.3 (R Core Team, 2019) or PLINK v1.90. The meta-analysis was performed in R version 3.6.3 (R core Team, 2019) using the metafor package.

**Supplementary Results**

*SNPs influence the change in DNA methylation during ICS treatment in GLUCOLD study.* Characteristics of patients are shown in Table S1. This analysis identified 76 inducible meQTL caused by 71 independent SNPs with an FDR < 0.05. The most significant association was between cg13086983 and rs10917023, where the G allele (MAF: 7.7%) induces higher methylation at this CpG site (Beta: 0.849, p-value: 4.21x10^-06^). The full list is represented in Table S2.

*Candidate-gene meta-analysis in PiCA.* A candidate-gene meta-analysis was performed with data from 1,515 European and 1,702 non-European children with asthma and treated with ICS from eight cohort studies using random effects. Summary characteristics of these patients are described in Table S3. The cohorts differed in asthma severity according to the percentages in the British Thoracic Society (BTS) treatment steps. In most cohorts, the majority of patients was treated according to BTS treatment step 2 (PACMAN, ESTATe, SAGE and Slovenia) and in two cohorts the majority was treated according to BTS treatment step 3 (SCSGES and PAGES). Variation was also seen in the percentage of the populations with ‘any exacerbations’, ranging from 9.9% in PACMAN to 58.5% in PAGES and 66.0% in GALA II. None of the identified meQTL were associated with the outcomes ‘any’ exacerbations and OCS courses in the past six to twelve months (Table S4-7).

*Candidate gene analysis in COPD:* A pharmacogenomic candidate gene association study for the genotype-by-ICS treatment effect on 3 years of FEV_1_ changes (estimated as slope) in 802 genotyped LHS-2 participants (Table S9).

**References**

1. van den Berge M, Steiling K, Timens W, Hiemstra PS, Sterk PJ, Heijink IH, Liu G, Alekseyev YO, Lenburg ME, Spira A, Postma DS. Airway gene expression in COPD is dynamic with inhaled corticosteroid treatment and reflects biological pathways associated with disease activity. *Thorax* 2014: 69(1): 14-23.

2. Nijnatten J, Brandsma C, Hiemstra P, Timens W, Van Den Berghe M, Faiz A. Changes in DNA methylation after corticosteroids treatment in COPD patients. European Respiratory Society International Congress, Virtual conference, 2020.

3. Allam VSRR, Faiz A, Lam M, Rathnayake SNH, Ditz B, Pouwels SD, Brandsma CA, Timens W, Hiemstra PS, Tew GW, Neighbors M, Grimbaldeston M, van den Berge M, Donnelly S, Phipps S, Bourke JE, Sukkar MB. RAGE and TLR4 differentially regulate airway hyperresponsiveness: Implications for COPD. *Allergy* 2020.

4. van den Berge M, Steiling K, Timens W, Hiemstra PS, Sterk PJ, Heijink IH, Liu G, Alekseyev YO, Lenburg ME, Spira A. Airway gene expression in COPD is dynamic with inhaled corticosteroid treatment and reflects biological pathways associated with disease activity. *Thorax* 2014: 69(1): 14-23.

5. Hernandez-Pacheco N, Farzan N, Francis B, Karimi L, Repnik K, Vijverberg SJ, Soares P, Schieck M, Gorenjak M, Forno E, Eng C, Oh SS, Pérez-Méndez L, Berce V, Tavendale R, Samedy LA, Hunstman S, Hu D, Meade K, Farber HJ, Avila PC, Serebrisky D, Thyne SM, Brigino-Buenaventura E, Rodriguez-Cintron W, Sen S, Kumar R, Lenoir M, Rodriguez-Santana JR, Celedón JC, Mukhopadhyay S, Potočnik U, Pirmohamed M, Verhamme KM, Kabesch M, Palmer CNA, Hawcutt DB, Flores C, Maitland-van der Zee AH, Burchard EG, Pino-Yanes M. Genome-wide association study of inhaled corticosteroid response in admixed children with asthma. *Clin Exp Allergy* 2019: 49(6): 789-798.

6. Obeidat Me, Faiz A, Li X, Van Den Berge M, Hansel NN, Joubert P, Hao K, Brandsma C-A, Rafaels N, Mathias R. The pharmacogenomics of inhaled corticosteroids and lung function decline in COPD. *European Respiratory Journal* 2019: 54(6).

7. Farzan N, Vijverberg SJ, Andiappan AK, Arianto L, Berce V, Blanca-López N, Bisgaard H, Bønnelykke K, Burchard EG, Campo P, Canino G, Carleton B, Celedón JC, Chew FT, Chiang WC, Cloutier MM, Daley D, Den Dekker HT, Dijk FN, Duijts L, Flores C, Forno E, Hawcutt DB, Hernandez-Pacheco N, de Jongste JC, Kabesch M, Koppelman GH, Manolopoulos VG, Melén E, Mukhopadhyay S, Nilsson S, Palmer CN, Pino-Yanes M, Pirmohamed M, Potočnik U, Raaijmakers JA, Repnik K, Schieck M, Sio YY, Smyth RL, Szalai C, Tantisira KG, Turner S, van der Schee MP, Verhamme KM, Maitland-van der Zee AH. Rationale and design of the multiethnic Pharmacogenomics in Childhood Asthma consortium. *Pharmacogenomics* 2017: 18(10): 931-943.

8. Law CW, Chen Y, Shi W, Smyth GK. voom: Precision weights unlock linear model analysis tools for RNA-seq read counts. *Genome Biol* 2014: 15(2): R29.

9. Palmer CN, Lipworth BJ, Lee S, Ismail T, Macgregor DF, Mukhopadhyay S. Arginine-16 beta2 adrenoceptor genotype predisposes to exacerbations in young asthmatics taking regular salmeterol. *Thorax* 2006: 61(11): 940-944.

10. Palmer CN, Doney AS, Lee SP, Murrie I, Ismail T, Macgregor DF, Mukhopadhyay S. Glutathione S-transferase M1 and P1 genotype, passive smoking, and peak expiratory flow in asthma. *Pediatrics* 2006: 118(2): 710-716.

11. Tavendale R, Macgregor DF, Mukhopadhyay S, Palmer CN. A polymorphism controlling ORMDL3 expression is associated with asthma that is poorly controlled by current medications. *J Allergy Clin Immunol* 2008: 121(4): 860-863.

12. Pino-Yanes M, Thakur N, Gignoux CR, Galanter JM, Roth LA, Eng C, Nishimura KK, Oh SS, Vora H, Huntsman S, Nguyen EA, Hu D, Drake KA, Conti DV, Moreno-Estrada A, Sandoval K, Winkler CA, Borrell LN, Lurmann F, Islam TS, Davis A, Farber HJ, Meade K, Avila PC, Serebrisky D, Bibbins-Domingo K, Lenoir MA, Ford JG, Brigino-Buenaventura E, Rodriguez-Cintron W, Thyne SM, Sen S, Rodriguez-Santana JR, Bustamante CD, Williams LK, Gilliland FD, Gauderman WJ, Kumar R, Torgerson DG, Burchard EG. Genetic ancestry influences asthma susceptibility and lung function among Latinos. *J Allergy Clin Immunol* 2015: 135(1): 228-235.

13. Turner SW, Ayres JG, Macfarlane TV, Mehta A, Mehta G, Palmer CN, Cunningham S, Adams T, Aniruddhan K, Bell C, Corrigan D, Cunningham J, Duncan A, Hunt G, Leece R, MacFadyen U, McCormick J, McLeish S, Mitra A, Miller D, Waxman E, Webb A, Wojcik S, Mukhopadhyay S, Macgregor D. A methodology to establish a database to study gene environment interactions for childhood asthma. *BMC Med Res Methodol* 2010: 10: 107.

14. Koster ES, Raaijmakers JA, Vijverberg SJ, Maitland-van der Zee AH. Inhaled corticosteroid adherence in paediatric patients: the PACMAN cohort study. *Pharmacoepidemiol Drug Saf* 2011: 20(10): 1064-1072.

15. White MJ, Risse-Adams O, Goddard P, Contreras MG, Adams J, Hu D, Eng C, Oh SS, Davis A, Meade K, Brigino-Buenaventura E, LeNoir MA, Bibbins-Domingo K, Pino-Yanes M, Burchard EG. Novel genetic risk factors for asthma in African American children: Precision Medicine and the SAGE II Study. *Immunogenetics* 2016: 68(6-7): 391-400.

**Table S1. Patient characteristics of the GLUCOLD study**

| **N** | **43** |
| --- | --- |
| **Age mean ± SD** | **61.1 ±6.9** |
| **Gender male n(%)** | **38 (90.5)** |
| **Smoking status n(%)** | **26 (61.9)** |
| **FEV_1_ (%predicted) mean ± SD** | **58.3 ± 10.3** |
| **FEV_1_/FVC mean ± SD** | **50.4 ±8.3** |

FEV_1_: forced expiratory volume in one second, FVC: forced vital capacity, N: number, SD: standard deviation.

**Table S2. Full list of identified inducible meQTL of bronchial biopsies of COPD patients in the GLUCOLD study**

| **SNPs** | **CpG site** | **statistic** | **p-value** | **FDR** | **beta** | **N SNPs tested** |
| --- | --- | --- | --- | --- | --- | --- |
| rs10917023 | cg13086983 | 5.366 | 4.21x10^-6^ | 7.66x10^-4^ | 0.849 | 182 |
| rs12310859 | cg02681173 | -5.474 | 3.00x10^-6^ | 9.15x10^-4^ | -1.034 | 305 |
| rs12316152 | cg17222500 | 4.954 | 1.53x10^-5^ | 1.19x10^-3^ | 1.079 | 78 |
| rs9859566 | cg08570199 | -4.680 | 3.59x10^-5^ | 3.09x10^-3^ | -0.695 | 120 |
| rs2399449 | cg08570199 | -4.563 | 5.15x10^-5^ | 3.09x10^-3^ | -0.690 | 120 |
| rs11039 | cg19563107 | -4.961 | 1.50x10^-5^ | 3.18x10^-3^ | -0.767 | 212 |
| rs7683170 | cg25805763 | -4.752 | 2.87x10^-5^ | 3.39x10^-3^ | -0.489 | 118 |
| rs6599223 | cg00782866 | -4.661 | 3.80x10^-5^ | 3.53x10^-3^ | -0.546 | 93 |
| rs854334 | cg12890903 | -4.852 | 2.11x10^-5^ | 3.86x10^-3^ | -0.573 | 183 |
| rs2172326 | cg02681173 | -4.612 | 4.42x10^-5^ | 6.74x10^-3^ | -0.993 | 305 |
| rs2129855 | cg11870591 | 4.682 | 3.57x10^-5^ | 6.75x10^-3^ | 0.751 | 189 |
| rs7342890 | cg26439733 | 4.228 | 1.43x10^-4^ | 6.94x10^-3^ | 0.651 | 146 |
| rs7220099 | cg26439733 | 4.228 | 1.43x10^-4^ | 6.94x10^-3^ | 0.651 | 146 |
| rs9896737 | cg26439733 | 4.228 | 1.43x10^-4^ | 6.94x10^-3^ | 0.651 | 146 |
| rs1849384 | cg17222500 | 4.142 | 1.85x10^-4^ | 7.21x10^-3^ | 0.868 | 78 |
| rs1019179 | cg23462990 | -4.692 | 3.45x10^-5^ | 7.90x10^-3^ | -0.872 | 229 |
| rs12890808 | cg12890903 | -4.356 | 9.68x10^-5^ | 8.86x10^-3^ | -0.530 | 183 |
| rs2320 | cg27367170 | 4.362 | 9.51x10^-5^ | 1.05x10^-2^ | 0.641 | 221 |
| rs633797 | cg27367170 | 4.362 | 9.51x10^-5^ | 1.05x10^-2^ | 0.641 | 221 |
| rs11605965 | cg12371372 | 4.218 | 1.47x10^-4^ | 1.21x10^-2^ | 0.712 | 82 |
| rs6586361 | cg24050135 | -4.332 | 1.04x10^-4^ | 1.60x10^-2^ | -0.911 | 154 |
| rs1862466 | cg00455402 | -4.304 | 1.13x10^-4^ | 1.72x10^-2^ | -0.449 | 152 |
| rs245183 | cg20744943 | -4.252 | 1.33x10^-4^ | 1.90x10^-2^ | -0.623 | 143 |
| rs7619506 | cg25150351 | 3.892 | 3.88x10^-4^ | 1.91x10^-2^ | 0.540 | 96 |
| rs12695746 | cg25150351 | 3.884 | 3.98x10^-4^ | 1.91x10^-2^ | 0.540 | 96 |
| rs241324 | cg05813995 | 4.037 | 2.53x10^-4^ | 1.93x10^-2^ | 0.404 | 76 |
| rs2833101 | cg26023019 | 4.012 | 2.73x10^-4^ | 1.96x10^-2^ | 0.990 | 204 |
| rs728174 | cg26023019 | 4.012 | 2.73x10^-4^ | 1.96x10^-2^ | 0.990 | 204 |
| rs2833117 | cg26023019 | 3.994 | 2.88x10^-4^ | 1.96x10^-2^ | 0.907 | 204 |
| rs10783203 | cg26698347 | -4.152 | 1.80x10^-4^ | 2.06x10^-2^ | -0.498 | 229 |
| rs7971524 | cg26698347 | -4.152 | 1.80x10^-4^ | 2.06x10^-2^ | -0.498 | 229 |
| rs4983025 | cg12890903 | -3.888 | 3.93x10^-4^ | 2.14x10^-2^ | -0.466 | 183 |
| rs12889256 | cg12890903 | -3.824 | 4.74x10^-4^ | 2.14x10^-2^ | -0.518 | 183 |
| rs7161311 | cg12890903 | -3.752 | 5.84x10^-4^ | 2.14x10^-2^ | -0.479 | 183 |
| rs2250243 | cg05732750 | -4.020 | 2.66x10^-4^ | 2.16x10^-2^ | -0.658 | 162 |
| rs872424 | cg05732750 | -4.020 | 2.66x10^-4^ | 2.16x10^-2^ | -0.658 | 162 |
| rs347684 | cg25150351 | 3.697 | 6.87x10^-4^ | 2.20x10^-2^ | 0.523 | 96 |
| rs241324 | cg06512918 | 4.013 | 2.72x10^-4^ | 2.20x10^-2^ | 0.441 | 81 |
| rs1806722 | cg16451661 | -4.004 | 2.79x10^-4^ | 2.26x10^-2^ | -0.494 | 81 |
| rs347685 | cg25150351 | 3.534 | 0.0011 | 2.27x10^-2^ | 0.478 | 96 |
| rs4683653 | cg25150351 | 3.507 | 0.0012 | 2.27x10^-2^ | 0.484 | 96 |
| rs832540 | cg16534315 | -4.001 | 2.82x10^-4^ | 2.33x10^-2^ | -0.315 | 248 |
| rs832535 | cg16534315 | -4.001 | 2.82x10^-4^ | 2.33x10^-2^ | -0.315 | 248 |
| rs252894 | cg16534315 | -4.001 | 2.82x10^-4^ | 2.33x10^-2^ | -0.315 | 248 |
| rs12465387 | cg12226731 | -4.351 | 9.85x10^-5^ | 2.36x10^-2^ | -0.637 | 240 |
| rs12636098 | cg18096987 | 4.172 | 1.69x10^-4^ | 2.37x10^-2^ | 0.700 | 140 |
| rs10784294 | cg21164131 | -4.104 | 2.07x10^-4^ | 2.42x10^-2^ | -0.594 | 117 |
| rs1791379 | cg10571908 | -4.336 | 1.03x10^-4^ | 2.59x10^-2^ | -0.616 | 251 |
| rs2681512 | cg20744943 | -3.915 | 3.63x10^-4^ | 2.60x10^-2^ | -0.590 | 143 |
| rs10861973 | cg24727480 | -4.203 | 1.54x10^-4^ | 2.62x10^-2^ | -0.608 | 170 |
| rs4821872 | cg02516101 | 4.225 | 1.44x10^-4^ | 2.70x10^-2^ | 0.646 | 187 |
| rs4315473 | cg19498266 | -3.952 | 3.25x10^-4^ | 2.755 x10^-2^ | -0.524 | 117 |
| rs7604580 | cg19498266 | -3.826 | 4.71x10^-4^ | 2.76x10^-2^ | -0.522 | 117 |
| rs796051 | cg13897977 | 3.987 | 2.93x10^-4^ | 3.11x10^-2^ | 0.570 | 106 |
| rs12310859 | cg00901320 | -4.305 | 1.13x10^-4^ | 3.26x10^-2^ | -0.552 | 288 |
| rs7608734 | cg00697965 | -4.039 | 2.52x10^-4^ | 3.31x10^-2^ | -0.582 | 202 |
| rs2701337 | cg00697965 | 3.950 | 3.27x10^-4^ | 3.31x10^-2^ | 0.607 | 202 |
| rs591510 | cg27367170 | 3.831 | 4.64x10^-4^ | 3.42x10^-2^ | 0.585 | 221 |
| rs4649058 | cg12459932 | 4.088 | 2.17x10^-4^ | 3.52x10^-2^ | 0.674 | 162 |
| rs2528584 | cg19498266 | -3.595 | 9.19x10^-4^ | 3.58x10^-2^ | -0.522 | 117 |
| rs4691233 | cg25805763 | -3.738 | 6.10x10^-4^ | 3.60x10^-2^ | -0.426 | 118 |
| rs13295426 | cg14406134 | -4.093 | 2.14x10^-4^ | 3.64x10^-2^ | -0.555 | 170 |
| rs2725236 | cg20462345 | 3.964 | 3.14x10^-4^ | 3.68x10^-2^ | 0.439 | 117 |
| rs7486109 | cg06003296 | -4.042 | 2.49x10^-4^ | 3.81x10^-2^ | -0.756 | 153 |
| rs12636098 | cg09299789 | -3.988 | 2.92x10^-4^ | 3.86x10^-2^ | -0.412 | 132 |
| rs12004778 | cg24248548 | 4.019 | 2.67x10^-4^ | 3.95x10^-2^ | 0.464 | 148 |
| rs11607961 | cg23710751 | 3.985 | 2.95x10^-4^ | 3.98x10^-2^ | 0.624 | 270 |
| rs11605822 | cg23710751 | 3.985 | 2.95x10^-4^ | 3.98x10^-2^ | 0.624 | 270 |
| rs11607961 | cg05931265 | 3.931 | 3.46x10^-4^ | 4.26x10^-2^ | 0.532 | 246 |
| rs11605822 | cg05931265 | 3.931 | 3.46x10^-4^ | 4.26x10^-2^ | 0.532 | 246 |
| rs10819242 | cg06603943 | 3.870 | 4.14x10^-4^ | 4.35x10^-2^ | 0.814 | 210 |
| rs10819246 | cg06603943 | 3.870 | 4.14x10^-4^ | 4.35x10^-2^ | 0.814 | 210 |
| rs2513081 | cg12371372 | 3.511 | 0.0012 | 4.79x10^-2^ | 0.577 | 82 |
| rs10760840 | cg06359101 | 3.547 | 0.0011 | 4.82x10^-2^ | 1.155 | 137 |
| rs10760846 | cg06359101 | 3.547 | 0.0011 | 4.82x10^-2^ | 1.155 | 137 |
| rs10820084 | cg06359101 | 3.547 | 0.0011 | 4.82x10^-2^ | 1.155 | 137 |

FDR: false discovery rate, N: number, SNPs: single nucleotide polymorphisms

**Table S3. Inducible eQTMs of bronchial biopsies of COPD patients in the GLUCOLD study**

| **CpG site** | **gene** | **symbol** | **beta** | **p-value** |
| --- | --- | --- | --- | --- |
| cg08570199 | ENSG00000091986 | CCDC80 | -1.249 | 2.049E-04 |
| cg26439733 | ENSG00000108349 | CASC3 | -0.366 | 1.591E-03 |
| cg26698347 | ENSG00000134291 | TMEM106C | -1.096 | 3.067E-03 |
| cg24727480 | ENSG00000198855 | FICD | 1.195 | 4.057E-03 |
| cg00782866 | ENSG00000230409 | TCEA1P2 | 1.205 | 5.177E-03 |
| cg00697965 | ENSG00000163359 | COL6A3 | -0.844 | 6.745E-03 |
| cg24248548 | ENSG00000173611 | SCAI | -0.600 | 6.806E-03 |
| cg06359101 | ENSG00000155827 | RNF20 | -0.188 | 1.234E-02 |
| cg26023019 | ENSG00000156284 | CLDN8 | -0.698 | 1.313E-02 |
| cg05813995 | ENSG00000143776 | CDC42BPA | -0.629 | 1.528E-02 |
| cg06603943 | ENSG00000136828 | RALGPS1 | -0.467 | 1.765E-02 |
| cg12459932 | ENSG00000117640 | MTFR1L | 0.281 | 2.159E-02 |
| cg12226731 | ENSG00000138385 | SSB | 0.486 | 2.703E-02 |
| cg24050135 | ENSG00000168264 | IRF2BP2 | -0.313 | 2.746E-02 |
| cg27367170 | ENSG00000134460 | IL2RA | 2.401 | 2.947E-02 |
| cg25150351 | ENSG00000114126 | TFDP2 | -0.640 | 3.083E-02 |
| cg06512918 | ENSG00000143776 | CDC42BPA | -0.473 | 3.953E-02 |
| cg19498266 | ENSG00000153237 | CCDC148 | -1.108 | 4.017E-02 |
| cg16451661 | ENSG00000058262 | SEC61A1 | 0.676 | 4.178E-02 |
| cg09299789 | ENSG00000144559 | TAMM41 | 0.448 | 4.380E-02 |
| cg02516101 | ENSG00000100311 | PDGFB | 0.947 | 4.465E-02 |
| cg12371372 | ENSG00000124942 | AHNAK | 0.407 | 4.522E-02 |
| cg00455402 | ENSG00000268357 | VN1R81P | 1.792 | 4.865E-02 |
| cg13086983 | ENSG00000075151 | EIF4G3 | -0.275 | 4.964E-02 |

**Table S4. Summary of the characteristics of children with asthma per cohort included in the meta-analysis**

|  | **PACMAN (n=704)** | **SCSGES (n=139)** | **BREATHE (n=113)** | **PAGES (n=422)** | **ESTATe (n=101)** | **GALA II (n=996)** | **SAGE (n=567)** | **Slovenia (n=175)** |
| --- | --- | --- | --- | --- | --- | --- | --- | --- |
| **Age (years), mean (SD)** | 8.8 (2.31) | 13.6 (5.26) | 7.9 (5.1) | 9.9 (3.53) | 10.4 (4.2) | 12.2 (3.2) | 13.6 (3.5) | 10.9 (3.4) |
| **Gender (female), % (n)** | 38.1 (268) | 29.5 (41) | 36.3 (41) | 41.9 (177) | 41.6 (42) | 43.1 (429) | 47.5 (270) | 45.1 (79) |
| **Family ethnicity, % (n)** |  |  |  |  |  |  |  |  |
| European | 90.8 (639) | 0 (0) | 0 (0) | 67.5 (285) | 94.0 (95) | 0 (0) | 0 (0) | 100 (95) |
| Mixed | 6.1 (43) | 0 (0) | 0 (0) | 0.5 (2) | 2.0 (2) | 0 (0) | 0 (0) | 0 (0) |
| Asian | 0.6 (4) | 100.0 (139) | 0 (0) | 2.1 (9) | 1.0 (1) | 0 (0) | 0 (0) | 0 (0) |
| African | 1.3 (9) | 0 (0) | 0 (0) | 0 (0) | 1.0 (1) | 0 (0) | 0 (0) | 0 (0) |
| Hispanic/Latino | 0.3 (2) | 0 (0) | 0 (0) | 0 (0) | 0 (0) | 100 (996) | 0 (0) | 0 (0) |
| African American | 0.0 (0) | 0 (0) | 0 (0) | 0 (0) | 0 (0) | 0 (0) | 100 (568) | 0 (0) |
| Unknown | 1.0 (7) | 0 (0) | 100 (113) | 29.9 (126) | 2.0 (2) | 0 (0) | 0 (0) | 0 (0) |
| **Atopy, % (n)** |  |  |  |  |  |  |  |  |
| Eczema | 65.3 (460) | 33.1 (46) | 61.9 (70) | 64.2 (271) | NA | 18.5 (184) | 40.6 (230) | 75.4 (132) |
| Food allergy | 50.1 (353) | NA | NA | 32.9 (139) | NA | NA | NA | NA |
| Allergic rhinitis | 43.8 (308) | 79.9 (111) | 57.5 (65) | 59.5 (251) | NA | 48.0 (478) | 55.7 (316) | 75.4 (132) |
| **Treatment group, % (n)** |  |  |  |  |  |  |  |  |
| **BTS step 2:** ICS + SABA | 70.0 (493) | 22.3 (31) | 42.5 (48) | 34.6 (146) | 64.3 (65) | 44.8 (446) | 67.2 (381) | 93.1 (163) |
| **BTS step 3:** ICS + SABA + LABA *or* ICS + SABA + LTRA | 23.6 (166) | 77.7 (108) | 30.1 (34) | 54.5 (230) | 33.7 (34) | 41.2 (410) | 25.7 (146) | 6.9 (12) |
| **BTS step 4**: ICS + SABA + LABA + LTRA | 6.4 (45) | NA | 24.8 (28) | 10.7 (45) | 2.0 (2) | 14.1 (140) | 7.0 (40) | 0 (0) |
| Other ICS regimen ** | 0 (0) | 0 (0) | 2.7 (3) | 0.2 (1) | 0 (0) | 0 (0) | 0 (0) | 0 (0) |
| **Asthma exacerbations, % (n)*** |  |  |  |  |  |  |  |  |
| ER visits | 5.4 (38) | 20.1 (28) | NA | NA | NA | 56.5 (563) | 44.6 (253) | 27.4 (48) |
| Hospitalisations | NA | 4.3 (6) | 14.2 (16) | 31.5 (113) | 12.9 (13) | 12.1 (121) | 5.8 (33) | 9.7 (17) |
| OCS use | 6.3 (44) | 20.1 (28) | 40.7 (46) | 52.6 (222) | 35.6 (36) | 39.6 (394) | 28.2 (160) | 12.6 (22) |
| Any exacerbations | 9.9 (70) | 33.8 (47) | 41.6 (47) | 58.5 (247) | 48.5 (49) | 66.0 (657) | 52.9 (300) | 33.7 (59) |
| **Uncontrolled asthma, % (n)** |  |  |  |  |  |  |  |  |
| ACQ > 0.75 | 41.5 (292) | NA | NA | NA | NA | NA | NA | NA |
| ACT ≤ 19 | NA | 3.6 (5) | NA | 36.5 (154) | NA | NA | NA | NA |
| **FeNO (ppb), mean (SD)** | 21.1 (29.9) | NA | NA | NA | NA | NA | NA | 46.7 (31.4) |

ACQ: asthma control questionnaire, ACT: asthma control test, BTS: British Thoracic Society, ER: emergency room, ESTATE: Effectiveness and Safety of Treatment with Asthma Therapy in children, FeNO: fraction of exhaled nitric oxide, GALA II: Genes-environments & Admixture in Latino Americans study, ICS: inhaled corticosteroids, LABA: long-acting beta2-agonists, LTRA: leukotriene receptor antagonists, NA: not available, OCS: oral corticosteroids, PACMAN: Pharmacogenetics of Asthma Medication in Children: Medication with ANti-inflammatory effects, PAGES: Paediatric Asthma Gene Environment Study, SABA: short-acting beta agonists, SAGE: Study of American Africans, Asthma Genes and Environments, SCGES: Singapore Cross Sectional Genetic Epidemiology Study, SD: standard deviation. Recent exacerbations refers to past 12 months, except for OCS usage, which refers to lifetime usage.

** Other ICS treatment regimen: Individuals who use ICS and therefore are included for the analysis but are not treated conform the BTS-steps.

**Table S5. Results of associations of the selected meQTL with exacerbations in European childhood asthma cohorts**

|  |  | **BREATHE/PAGES (n=535)** | | **PACMAN (n=704)** | | **ESTATE (n=101)** | | **Slovenia (n=175)** | | **Overall** |  |
| --- | --- | --- | --- | --- | --- | --- | --- | --- | --- | --- | --- |
| **SNP** | **Allele (R/E)** | **OR (95% CI)** | **Allele frequency** | **OR (95% CI)** | **Allele frequency** | **OR (95%CI)** | **Allele frequency** | **OR (95% CI)** | **Allele frequency** | **OR (95% CI)** | **p-value** |
| rs241324 | G/A | 0.85 (0.59-1.23) | 0.16 | 0.95 (0.60-1.45) | 0.19 | 0.61 (0.25-1.43) | 0.84 | 1.31 (0.67-2.56) | 0.15 | 0.91 (0.71-1.17) | 0.47 |
| rs6586361 | T/G | 0.84 (0.63-1.11) | 0.47 | 0.91 (0.64-1.30) | 0.49 | 1.06 (0.56-2.00) | 0.46 | 1.29 (0.79-2.09) | 0.50 | 0.94 (0.77-1.14) | 0.52 |
| rs7604580 | G/A | 1.07 (0.80-1.44) | 0.30 | 0.81 (0.42-1.57) | 0.73 | 1.46 (0.72-2.98) | 0.31 | 1.18 (0.72-1.93) | 0.30 | 1.09 (0.87-1.37) | 0.45 |
| rs4315473 | C/T | 1.09 (0.80-1.49) | 0.27 | 1.05 (0.52-2.04) | 0.27 | 1.28 (0.61-2.70) | 0.73 | 1.10 (0.66-1.82) | 0.28 | 1.11 (0.88-1.40) | 0.40 |
| rs12465387 | G/A | 0.88 (0.54-1.07) | 0.08 | 1.07 (0.53-1.96) | 0.07 | 1.24 (0.41-3.85) | 0.10 | 1.02 (0.43-2.40) | 0.08 | 0.98 (0.70-1.38) | 0.92 |
| rs7608734 | G/A | 0.76 (0.54-1.07) | 0.20 | 0.93 (0.69-1.27) | 0.68 | 0.81 (0.40-1.63) | 0.28 | 1.03 (0.53-2.02) | 0.15 | 0.86 (0.70-1.06) | 0.16 |
| rs2701337 | G/A | 0.79 (0.59-1.06) | 0.40 | 1.45 (0.89-2.41) | 0.76 | 1.02 (0.59-1.79) | 0.58 | 0.84 (0.53-1.33) | 0.40 | 0.95 (0.73-1.11) | 0.69 |
| rs12636098 | C/T | 1.27 (0.92-1.77) | 0.24 | 0.85 (0.59-1.25) | 0.72 | 0.57 (0.27-1.15) | 0.25 | 0.79 (0.47-1.34) | 0.27 | 0.91 (0.67-1.24) | 0.56 |
| rs2129855 | T/C | 1.15 (0.87-1.51) | 0.45 | 1.00 (0.70-1.44) | 0.50 | 0.85 (0.44-1.6) | 0.47 | 1.23 (0.78-1.93) | 0.47 | 1.09 (0.90-1.32) | 0.36 |
| rs6599223 | C/T | 1.13 (0.79-1.63) | 0.17 | 1.01 (0.61-1.62) | 0.16 | 3.13 (1.32-8.33) | 0.83 | 1.14 (0.62-2.12) | 0.15 | 1.27 (0.84-1.94) | 0.26 |
| rs9859566 | A/G | 0.96 (0.70-1.31) | 0.27 | 0.82 (0.55-1.19) | 0.29 | 0.75 (0.38-1.44) | 0.31 | 1.02 (0.60-1.72) | 0.30 | 0.90 (0.73-1.11) | 0.34 |
| rs2399449 | T/C | 0.94 (0.71-1.24) | 0.37 | 0.87 (0.61-1.22) | 0.40 | 0.76 (0.40-1.43) | 0.66 | 0.94 (0.58-1.53) | 0.37 | 0.90 (0.74-1.09) | 0.27 |
| rs1806722 | T/C | 1.12 (0.81-1.56) | 0.24 | 1.16 (0.75-1.59) | 0.21 | 1.86 (0.87-4.12) | 0.20 | 1.19 (0.66-2.12) | 0.21 | 1.19 (0.95-1.49) | 0.13 |
| rs12695746 | G/T | 0.91 (0.62-1.34) | 0.16 | 1.02 (0.63-1.58) | 0.16 | 0.99 (0.46-2.13) | 0.82 | 0.53 (0.26-1.08) | 0.15 | 0.89 (0.69-1.15) | 0.36 |
| rs4683653 | T/C | 0.96 (0.71-1.29) | 0.32 | 1.08 (0.75-1.59) | 0.68 | 0.90 (0.47-1.72) | 0.34 | 1.03 (0.62-1.73) | 0.30 | 1.00 (0.82-1.22) | 1.00 |
| rs347684 | C/T | 1.06 (0.73-1.55) | 0.15 | 0.78 (0.44-1.31) | 0.14 | 0.79 (0.35-1.72) | 0.83 | 0.52 (0.25-1.08) | 0.15 | 0.86 (0.65-1.14) | 0.29 |
| rs347685 | A/C | 1.10 (0.81-1.43) | 0.29 | 0.76 (0.50-1.13) | 0.28 | 1.15 (0.62-2.17) | 0.73 | 0.81 (0.47-1.38) | 0.29 | 0.96 (0.78-1.18) | 0.68 |
| rs7683170 | C/A | 1.01 (0.77-1.34) | 0.32 | 0.91 (0.60-1.35) | 0.28 | 1.31 (0.66-2.63) | 0.28 | 1.50 (0.88-2.56) | 0.25 | 1.06 (0.87-1.30) | 0.54 |
| rs832540 | A/G | 1.07 (0.81-1.43) | 0.37 | 1.14 (0.79-1.64) | 0.60 | 1.30 (0.70-2.44) | 0.61 | 0.61 (0.36-1.03) | 0.36 | 1.01 (0.76-1.34) | 0.96 |
| rs832535 | T/C | 1.08 (0.81-1.43) | 0.37 | 0.97 (0.68-1.38) | 0.41 | 1.41 (0.76-2.63) | 0.60 | 0.76 (0.46-1.24) | 0.37 | 1.02 (0.84-1.23) | 0.86 |
| rs252894 | A/G | 1.06 (0.80-1.41) | 0.37 | 1.01 (0.71-1.44) | 0.59 | 1.41 (0.76-2.63) | 0.60 | 0.76 (0.46-1.24) | 0.37 | 1.02 (0.84-1.24) | 0.82 |
| rs245183 | T/G | 0.91 (0.67-1.22) | 0.29 | 1.16 (0.79-1.74) | 0.71 | 1.64 (0.79-3.57) | 0.72 | 1.37 (0.86-2.20) | 0.32 | 1.11 (0.89-1.40) | 0.36 |
| rs2681512 | A/G | 0.93 (0.69-1.24) | 0.30 | 0.79 (0.53-1.15) | 0.32 | 1.89 (0.98-3.85) | 0.69 | 1.68 (1.02-2.75) | 0.32 | 1.16 (0.77-1.75) | 0.48 |
| rs2250243 | T/C | 0.93 (0.69-1.24) | 0.28 | 1.16 (0.77-1.71) | 0.23 | 0.85 (0.40-1.79) | 0.24 | 0.72 (0.42-1.25) | 0.25 | 0.95 (0.77-1.16) | 0.60 |
| rs872424 | C/A | 0.94 (0.71-1.26) | 0.27 | 1.18 (0.79-1.74) | 0.23 | 0.82 (0.38-1.74) | 0.24 | 0.67 (0.39-1.15) | 0.25 | 0.95 (0.77-1.16) | 0.60 |
| rs1019179 | A/G | 0.74 (0.53-1.02) | 0.24 | 0.82 (0.51-1.26) | 0.23 | 1.23 (0.60-2.52) | 0.23 | 1.12 (0.69-1.80) | 0.28 | 0.87 (0.70-1.08) | 0.20 |
| rs10760840 | G/A | 1.08 (0.60-1.98) | 0.06 | 1.70 (0.91-3.03) | 0.07 | 0.46 (0.09-2.25) | 0.05 | 1.06 (0.36-3.17) | 0.05 | 1.24 (0.84-1.82) | 0.28 |
| rs10760846 | T/C | 1.23 (0.68-2.30) | 0.06 | 1.82 (0.98-3.25) | 0.07 | 0.30 (0.05-1.61) | 0.04 | 1.17 (0.38-3.55) | 0.04 | 1.25 (0.73-1.80) | 0.41 |
| rs10820084 | C/T | 1.13 (0.65-1.99) | 0.07 | 2.16 (1.26-3.62) | 0.08 | 0.88 (0.27-2.65) | 0.08 | 0.80 (0.27-2.40) | 0.06 | 1.34 (0.85-2.09) | 0.20 |
| rs13295426 | A/C | 1.17 (0.73-1.90) | 0.09 | 1.10 (0.56-1.99) | 0.08 | 0.37 (0.11-1.06) | 0.10 | 2.51 (1.12-5.65) | 0.08 | 1.12 (0.56-2.25) | 0.75 |
| rs12004778 | G/A | 1.21 (0.84-1.76) | 0.16 | 1.27 (0.78-2.01) | 0.15 | 1.53 (0.42-5.72) | 0.15 | 0.95 (0.55-1.63) | 0.21 | 1.17 (0.91-1.51) | 0.21 |
| rs2320 | T/G | 0.95 (0.72-1.24) | 0.43 | 1.10 (0.77-1.57) | 0.37 | 2.44 (1.23-5.17) | 0.40 | 0.84 (0.51-1.37) | 0.40 | 1.13 (0.75-1.70) | 0.56 |
| rs591510 | T/G | 0.94 (0.72-1.24) | 0.43 | 1.02 (0.71-1.46) | 0.37 | 2.72 (1.31-6.09) | 0.41 | 0.80 (0.49-1.31) | 0.40 | 1.12 (0.69-1.81) | 0.65 |
| rs633797 | G/A | 1.00 (0.76-1.31) | 0.43 | 1.04 (0.72-1.47) | 0.37 | 2.81 (1.28-6.66) | 0.41 | 0.85 (0.53-1.39) | 0.40 | 1.15 (0.73-1.80) | 0.55 |
| rs11605965 | C/T | 1.09 (0.71-1.67) | 0.11 | 1.48 (0.86-2.43) | 0.11 | 0.35 (0.11-1.01) | 0.11 | 0.90 (0.48-1.67) | 0.16 | 0.97 (0.58-1.60) | 0.89 |
| rs10783203 | G/A | 0.86 (0.64-1.16) | 0.33 | 1.01 (0.69-1.46) | 0.30 | 1.33 (0.68-2.63) | 0.69 | 1.00 (0.61-1.62) | 0.29 | 0.96 (0.78-1.17) | 0.68 |
| rs7971524 | G/T | 0.86 (0.64-1.16) | 0.33 | 1.01 (0.69-1.46) | 0.30 | 1.43 (0.73-2.84) | 0.68 | 0.97 (0.60-1.58) | 0.29 | 0.96 (0.79-1.17) | 0.70 |
| rs10784294 | G/A | 0.96 (0.73-1.27) | 0.39 | 0.96 (0.67-1.36) | 0.40 | 1.33 (0.74-2.44) | 0.57 | 0.91 (0.56-1.48) | 0.38 | 0.98 (0.81-1.19) | 0.87 |
| rs123165152 | A/C | 1.18 (0.66-2.14) | 0.06 | 1.78 (1.01-3.00) | 0.08 | 0.87 (0.21-3.57) | 0.05 |  |  | 1.41 (0.96-2.08) | 0.08 |
| rs1849384 | A/C | 1.03 (0.64-1.67) | 0.09 | 1.12 (0.67-1.80) | 0.12 | 1.90 (0.51-7.78) | 0.06 | 1.35 (0.47-3.92) | 0.06 | 1.13 (0.82-1.56) | 0.45 |
| rs10861973 | G/A | 1.09 (0.83-1.44) | 0.52 | 0.99 (0.70-1.40) | 0.48 | 0.95 (0.46-1.95) | 0.46 | 0.65 (0.39-1.09) | 0.46 | 0.98 (0.81-1.18) | 0.82 |
| rs12310859 | T/C | 1.00 (0.75-1.34) | 0.36 | 1.31 (0.90-1.88) | 0.38 | 0.88 (0.46-1.68) | 0.40 | 1.05 (0.63-1.75) | 0.38 | 1.08 (0.88-1.31) | 0.46 |
| rs2172326 | C/T | 0.94 (0.68-1.30) | 0.23 | 1.17 (0.77-1.74) | 0.24 | 1.01 (0.49-2.10) | 0.25 | 1.03 (0.61-1.76) | 0.27 | 1.02 (0.82-1.28) | 0.83 |
| rs7161311 | A/G | 0.96 (0.72-1.29) | 0.36 | 0.97 (0.68-1.36) | 0.37 | 1.14 (0.58-2.29) | 0.32 | 0.67 (0.42-1.05) | 0.36 | 0.91 (0.75-1.11) | 0.36 |
| rs12890808 | G/A | 0.92 (0.69-1.23) | 0.39 | 0.98 (0.69-1.37) | 0.38 | 0.91 (0.45-1.85) | 0.42 | 0.86 (0.52-1.43) | 0.38 | 0.93 (0.76-1.13) | 0.46 |
| rs4983025 | T/C | 0.99 (0.72-1.38) | 0.22 | 0.93 (0.60-1.38) | 0.23 | 2.54 (1.18-5.78) | 0.24 | 0.75 (0.44-1.30) | 0.26 | 1.07 (0.68-1.71) | 0.76 |
| rs12889256 | T/C | 0.94 (0.70-1.27) | 0.33 | 1.03 (0.72-1.46) | 0.33 | 1.75 (0.88-3.61) | 0.30 | 0.77 (0.48-1.25) | 0.35 | 1.00 (0.77-1.28) | 0.97 |
| rs11039 | T/G | 1.50 (0.97-2.34) | 0.12 | 0.89 (0.52-1.45) | 0.15 | 0.97 (0.43-2.15) | 0.18 | 1.39 (0.67-2.88) | 0.13 | 1.20 (0.90-1.59) | 0.21 |
| rs7342890 | T/C | 1.12 (0.79-1.59) | 0.19 | 1.24 (0.78-1.93) | 0.17 | 0.98 (0.49-1.96) | 0.75 | 1.25 (0.68-2.30) | 0.18 | 1.15 (0.91-1.46) | 0.23 |
| rs7220099 | A/G | 1.05 (0.74-1.49) | 0.19 | 1.28 (0.82-1.94) | 0.18 | 0.98 (0.49-1.96) | 0.75 | 1.18 (0.64-2.17) | 0.19 | 1.12 (0.89-1.42) | 0.33 |
| rs9896737 | G/A | 1.05 (0.74-1.49) | 0.19 | 1.26 (0.80-1.92) | 0.18 | 0.98 (0.49-1.96) | 0.75 | 1.18 (0.64-2.17) | 0.19 | 1.12 (0.88-1.41) | 0.35 |
| rs1791379 | C/A | 1.04 (0.76-1.44) | 0.26 | 1.10 (0.72-1.64) | 0.21 | 1.15 (0.44-3.03) | 0.74 | 1.11 (0.65-1.88) | 0.26 | 1.08 (0.86-1.34) | 0.52 |
| rs1862466 | G/A | 0.95 (0.71-1.26) | 0.48 | 0.88 (0.62-1.24) | 0.48 | 0.81 (0.43-1.47) | 0.49 | 0.85 (0.54-1.33) | 0.45 | 0.89 (0.74-1.08) | 0.25 |
| rs2833101 | G/A | 1.00 (0.76-1.31) | 0.49 | 1.04 (0.73-1.49) | 0.49 | 1.15 (0.62-2.16) | 0.54 | 0.79 (0.50-1.25) | 0.49 | 0.98 (0.82-1.18) | 0.86 |
| rs728174 | C/T | 1.02 (0.78-1.33) | 0.48 | 1.02 (0.72-1.46) | 0.46 | 1.17 (0.63-2.18) | 0.53 | 0.80 (0.50-1.27) | 0.49 | 0.99 (0.82-1.20) | 0.94 |
| rs2833117 | A/C | 1.21 (0.92-1.60) | 0.42 | 1.02 (0.71-1.45) | 0.41 | 1.12 (0.62-2.04) | 0.45 | 0.99 (0.63-1.56) | 0.42 | 1.11 (0.92-1.34) | 0.29 |
| rs4821872 | C/T | 0.89 (0.67-1.19) | 0.32 | 1.07 (0.73-1.54) | 0.30 | 0.62 (0.32-1.18) | 0.33 | 1.30 (0.76-2.23) | 0.31 | 0.96 (0.76-1.21) | 0.73 |

CI: confidence interval, E: effect allele, ER: emergency room, ESTATE: Effectiveness and Safety of Treatment with Asthma Therapy in children, OCS: oral corticosteroids; OR: odds ratio for the effect alleles, PACMAN: Pharmacogenetics of Asthma Medication in Children: Medication with Anti-inflammatory effects, PAGES: Paediatric Asthma Gene Environment Study, NA: no individuals in at least one comparison group, R: reference allele, SNP: single nucleotide polymorphism. All results were corrected for age, gender and principal components

**Table S6. Results of associations of the selected meQTL with exacerbations in non-European childhood asthma cohorts**

|  |  | **SCSGES (n=139)** | | **SAGE (n=567)** | | **GALA II (n=996)** | | **Overall** |  |
| --- | --- | --- | --- | --- | --- | --- | --- | --- | --- |
| **SNP** | **Allele**  **(R/E)** | **OR (95% CI)** | **Allele frequency** | **OR (95% CI)** | **Allele frequency** | **OR (95% CI)** | **Allele frequency** | **OR (95% CI)** | **P-value** |
| rs241324 | A/G | 1.43 (0.78-2.61) | 0.56 | 1.04 (0.81-1.35) | 0.70 | 1.04 (0.82-1.31) | 0.77 | 1.06 (0.90-1.26) | 0.46 |
| rs6586361 | G/T | 2.17 (1.10-4.27) | 0.19 | 0.97 (0.74-1.25) | 0.67 | 1.24 (1.00-1.55) | 0.37 | 1.24 (0.88-1.74) | 0.22 |
| rs7604580 | G/A | 0.48 (0.21-1.06) | 0.17 | 0.76 (0.57-1.02) | 0.21 | 0.94 (0.73-1.20) | 0.23 | 0.82 (0.66-1.02) | 0.07 |
| rs4315473 | T/C | 1.60 (0.56-4.54) | 0.91 | 1.01 (0.79-1.27) | 0.48 | 1.01 (0.79-1.29) | 0.71 | 1.02 (0.86-1.21) | 0.82 |
| rs12465387 | G/A | 1.09 (0.56-2.15) | 0.17 | 1.30 (0.57-2.97) | 0.02 | 0.89 (0.63-1.27) | 0.09 | 0.97 (0.73-1.30) | 0.86 |
| rs7608734 | A/G | 1.66 (0.78-3.55) | 0.80 | 1.10 (0.86-1.41) | 0.55 | 0.91 (0.71-1.16) | 0.18 | 1.05 (1.00-1.10) | 0.06 |
| rs2701337 | A/G | 1.61 (0.85-3.04) | 0.76 | 0.89 (0.69-1.15) | 0.69 | 1.14 (0.93-1.39) | 0.59 | 1.07 (0.85-1.36) | 0.55 |
| rs12636098 | C/T | 0.76 (0.42-1.38) | 0.25 | 1.03 (0.78-1.42) | 0.26 | 0.95 (0.76-1.19) | 0.27 | 0.96 (0.81-1.14) | 0.66 |
| rs2129855 | T/C | 0.91 (0.51-1.61) | 0.36 | 1.05 (0.78-1.42) | 0.79 | 0.76 (0.62-0.94) | 0.54 | 0.88 (0.69-1.12) | 0.28 |
| rs6599223 | T/C | 0.77 (0.43-1.38) | 0.71 | 0.89 (0.65-1.22) | 0.81 | 0.98 (0.72-1.32) | 0.87 | 0.91 (0.74-1.12) | 0.37 |
| rs9859566 | A/G | 0.69 (0.30-1.62) | 0.56 | 1.06 (0.82-1.38) | 0.32 | 1.10 (0.88-1.38) | 0.29 | 1.06 (0.90-1.26) | 0.47 |
| rs2399449 | C/T | 0.90 (0.51-1.56) | 0.81 | 0.84 (0.63-1.14) | 0.20 | 1.01 (0.83-1.22) | 0.50 | 0.95 (0.81-1.11) | 0.51 |
| rs1806722 | T/C | 1.16 (0.53-2.52) | 0.13 | 0.91 (0.67-1.26) | 0.18 | 1.12 (0.88-1.43) | 0.22 | 1.05 (0.87-1.26) | 0.63 |
| rs12695746 | T/G | 0.85 (0.35-2.04) | 0.90 | 0.98 (0.58-1.65) | 0.94 | 1.17 (0.88-1.56) | 0.87 | 1.10 (0.87-1.40) | 0.44 |
| rs4683653 | T/C | 1.04 (0.56-1.94) | 0.26 | 0.99 (0.78-1.27) | 0.56 | 0.95 (0.76-1.18) | 0.33 | 0.97 (0.83-1.14) | 0.72 |
| rs347684 | T/C | 0.83 (0.41-1.67) | 0.82 | 0.81 (0.41-1.58) | 0.97 | 1.05 (0.75-1.45) | 0.90 | 0.97 (0.74-1.27) | 0.81 |
| rs347685 | C/A | 1.13 (0.62-2.08) | 0.71 | 1.11 (0.84-1.47) | 0.76 | 1.09 (0.85-1.40) | 0.78 | 1.10 (0.92-1.32) | 0.29 |
| rs7683170 | C/A | 1.02 (0.57-1.68) | 0.46 | 1.01 (0.65-1.57) | 0.09 | 0.93 (0.75-1.16) | 0.28 | 0.96 (0.79-1.15) | 0.63 |
| rs832540 | G/A | 0.57 (0.16-1.98) | 0.06 | 0.92 (0.69-1.22) | 0.24 | 0.84 (0.69-1.03) | 0.36 | 0.86 (0.73-1.02) | 0.08 |
| rs832535 | C/T | 0.57 (0.16-1.98) | 0.06 | 1.13 (0.88-1.44) | 0.38 | 0.80 (0.65-0.98) | 0.39 | 0.92 (0.67-1.25) | 0.58 |
| rs252894 | G/T | 0.57 (0.50-6.21) | 0.06 | 0.85 (0.63-1.15) | 0.20 | 0.84 (0.68-1.04) | 0.35 | 0.84 (0.71-0.99) | 0.04 |
| rs245183 | G/A | 1.43 (0.82-2.49) | 0.56 | 0.97 (0.76-1.24) | 0.63 | 1.04 (0.85-1.27) | 0.62 | 1.04 (0.89-1.20) | 0.63 |
| rs2681512 | G/T | 1.46 (0.85-2.51) | 0.52 | 0.91 (0.71-1.16) | 0.63 | 1.05 (0.87-1.28) | 0.60 | 1.02 (0.88-1.19) | 0.76 |
| rs2250243 | T/C | 0.42 (0.23-0.78) | 0.67 | 0.95 (0.68-1.31) | 0.16 | 0.95 (0.76-1.18) | 0.32 | 0.78 (0.50-1.21) | 0.27 |
| rs872424 | C/A | 0.50 (0.27-0.92) | 0.64 | 0.89 (0.65-1.22) | 0.18 | 0.97 (0.78-1.21) | 0.32 | 0.85 (0.66-1.10) | 0.23 |
| rs1019179 | A/G | 1.13 (0.46-1.70) | 0.25 | 1.38 (0.97-1.94) | 0.14 | 1.00 (0.81-1.24) | 0.28 | 1.13 (0.89-1.43) | 0.33 |
| rs10760840 | G/A | 1.12 (0.64-1.98) | 0.65 | 1.21 (0.84-1.76) | 0.13 | 0.97 (0.75-1.25) | 0.18 | 1.05 (0.86-1.28) | 0.62 |
| rs10760846 | T/C | 1.40 (0.83-2.37) | 0.56 | 1.25 (0.85-1.82) | 0.12 | 0.96 (0.74-1.25) | 0.16 | 1.11 (0.88-1.41) | 0.38 |
| rs10820084 | C/T | 1.25 (0.75-2.08) | 0.57 | 1.38 (0.84-2.28) | 0.06 | 1.00 (0.78-1.30) | 0.16 | 1.10 (0.89-1.36) | 0.37 |
| rs13295426 | A/C | 0.71 (0.21-2.35) | 0.06 | 0.74 (0.41-1.35) | 0.04 | 0.83 (0.54-1.29) | 0.05 | 0.79 (0.57-1.11) | 0.18 |
| rs12004778 | G/A | 1.37 (0.65-2.90) | 0.17 | 0.95 (0.69-1.30) | 0.19 | 0.96 (0.76-1.22) | 0.22 | 0.98 (0.81-1.18) | 0.82 |
| rs2320 | T/G | 1.00 (0.60-1.68) | 0.64 | 1.55 (1.15-2.09) | 0.21 | 0.86 (0.70-1.05) | 0.44 | 1.10 (0.75-1.61) | 0.63 |
| rs591510 | A/G | 0.99 (0.59-1.66) | 0.64 | 1.72 (1.20-2.45) | 0.15 | 0.84 (0.69-1.03) | 0.43 | 1.12 (0.72-1.74) | 0.62 |
| rs633797 | G/A | 1.05 (0.62-1.76) | 0.64 | 1.45 (1.04-2.03) | 0.17 | 0.84 (0.69-1.03) | 0.43 | 1.07 (0.75-1.52) | 0.72 |
| rs11605965 | C/T | 1.32 (0.61-2.85) | 0.16 | 1.14 (0.54-2.43) | 0.03 | 1.26 (0.82-1.95) | 0.08 | 1.25 (0.89-1.75) | 0.20 |
| rs10783203 | A/G | 0.74 (0.42-1.31) | 0.54 | 1.04 (0.79-1.35) | 0.70 | 1.06 (0.87-1.30) | 0.54 | 1.02 (0.88-1.20) | 0.76 |
| rs7971524 | T/C | 0.71 (0.40-1.25) | 0.54 | 1.02 (0.77-1.35) | 0.71 | 1.08 (0.88-1.32) | 0.55 | 1.08 (0.92-1.27) | 0.75 |
| rs10784294 | A/G | 1.26 (0.74-2.14) | 0.66 | 1.09 (0.87-1.37) | 0.53 | 1.00 (0.81-1.23) | 0.57 | 1.02 (0.88-1.18) | 0.83 |
| rs123165152 | A/C | NA |  | NA |  | NA |  |  |  |
| rs1849384 | A/C | 1.91 (0.25-1.08) | 0.18 | 1.08 (0.82-1.43) | 0.74 | 0.79 (0.59-1.07) | 0.20 | 1.07 (0.72-1.59) | 0.75 |
| rs10861973 | G/A | NA |  | 1.15 (0.88-1.49) | 0.65 | 0.88 (0.71-1.10) | 0.65 | 1.00 (0.77-1.29) | 0.97 |
| rs12310859 | T/C | 0.60 (0.32-1.13) | 0.27 | 1.25 (0.95-1.65) | 0.27 | 0.93 (0.74-1.18) | 0.25 | 0.97 (0.69-1.35) | 0.84 |
| rs2172326 | C/T | 0.67 (0.34-1.31) | 0.22 | 1.11 (0.75-1.65) | 0.11 | 1.10 (0.83-1.46) | 0.16 | 1.05 (0.84-1.30) | 0.68 |
| rs7161311 | T/G | 2.07 (1.13-3.77) | 0.37 | 1.16 (0.90-1.48) | 0.53 | 1.05 (0.84-1.31) | 0.33 | 1.17 (0.96-1.43) | 0.13 |
| rs12890808 | G/A | 1.19 (0.68-2.08) | 0.31 | 0.93 (0.73-1.18) | 0.58 | 0.93 (0.74-1.16) | 0.31 | 0.95 (0.81-1.11) | 0.49 |
| rs4983025 | T/C | NA |  | 0.80 (0.61-1.05) | 0.29 | 1.05 (0.79-1.40) | 0.17 | 0.91 (0.70-1.19) | 0.50 |
| rs12889256 | T/C | 1.12 (0.59-2.15) | 0.21 | 0.76 (0.59-0.97) | 0.43 | 0.86 (0.68-1.08) | 0.29 | 0.83 (0.70-0.97) | 0.02 |
| rs11039 | T/G | 0.81 (0.35-1.85) | 0.12 | 1.00 (0.57-1.74) | 0.05 | 1.00 (0.72-1.37) | 0.11 | 0.98 (0.75-1.27) | 0.85 |
| rs7342890 | C/T | 0.80 (0.30-2.13) | 0.92 | 1.12 (0.85-1.48) | 0.75 | 0.83 (0.67-1.02) | 0.69 | 0.94 (0.72-1.21) | 0.61 |
| rs7220099 | G/A | 0.78 (0.33-1.86) | 0.91 | 1.06 (0.83-1.34) | 0.43 | 0.90 (0.73-1.10) | 0.63 | 0.95 (0.82-1.11) | 0.55 |
| rs9896737 | A/G | 0.76 (0.29-1.96) | 0.92 | 1.07 (0.84-1.37) | 0.53 | 0.87 (0.71-1.08) | 0.65 | 0.95 (0.79-1.14) | 0.56 |
| rs1791379 | A/G | 0.99 (0.54-1.82) | 0.76 | 0.91 (0.53-1.55) | 0.95 | 0.90 (0.68-1.20) | 0.83 | 0.92 (0.73-1.16) | 0.46 |
| rs1862466 | A/G | 0.79 (0.47-1.34) | 0.63 | 0.96 (0.74-1.25) | 0.30 | 0.97 (0.79-1.20) | 0.42 | 0.95 (0.82-1.11) | 0.53 |
| rs2833101 | G/A | 1.47 (0.79-2.71) | 0.71 | 0.91 (0.69-1.19) | 0.68 | 1.03 (0.81-1.30) | 0.64 | 1.01 (0.85-1.19) | 0.95 |
| rs728174 | C/T | 1.25 (0.73-2.16) | 0.42 | 1.01 (0.77-1.32) | 0.32 | 1.10 (0.87-1.38) | 0.57 | 1.08 (0.91-1.27) | 0.39 |
| rs2833117 | A/C | 1.22 (0.70-2.14) | 0.44 | 1.17 (0.91-1.50) | 0.45 | 0.97 (0.78-1.20) | 0.57 | 1.07 (0.91-1.25) | 0.45 |
| rs4821872 | C/T | 1.08 (0.52-2.27) | 0.17 | 1.04 (0.76-1.43) | 0.16 | 1.01 (0.81-1.25) | 0.29 | 1.02 (0.86-1.22) | 0.80 |

CI: confidence interval, ER: emergency room, GALA II: Genes-environments & Admixture in Latino Americans study, NA: no individuals in at least one comparison group, OCS: oral corticosteroids, OR: odds ratio, SAGE: Study of African Americans, Asthma, Genes and Environments, SCSGES: Singapore Cross Sectional Genetic Epidemiology Study, SNP: single nucleotide polymorphism. All results were corrected for age, gender and principal components.

**Table S7. Results of associations of the selected meQTL with oral corticosteroid use in European childhood asthma cohorts**

|  |  | **BREATHE/PAGES (n=535)** | | **PACMAN (n=704)** | | **ESTATE (n=101)** | | **SLOVENIA (n=175)** | | **Overall** |  |
| --- | --- | --- | --- | --- | --- | --- | --- | --- | --- | --- | --- |
| **SNP** | **Allele (R/E)** | **OR**  **(95% CI)** | **Allele frequency** | **OR**  **(95% CI)** | **Allele frequency** | **OR (95% CI)** | **Allele frequency** | **OR**  **(95% CI)** | **Allele frequency** | **OR**  **(95% CI)** | **P-value** |
| rs241324 | G/A | 0.86 (0.57-1.27) | 0.16 | 0.97 (0.54-1.63) | 0.19 | 0.97 (0.34-2.62) | 0.84 | 1.02 (0.38-2.74) | 0.15 | 0.91 (0.68-1.22) | 0.54 |
| rs6586361 | T/G | 0.75 (0.56-1.02) | 0.47 | 0.96 (0.62-1.50) | 0.49 | 1.03 (0.50-2.16) | 0.46 | 1.32 (0.68-2.57) | 0.50 | 0.89 (0.70-1.14) | 0.36 |
| rs7604580 | G/A | 1.05 (0.77-1.43) | 0.30 | 0.58 (0.26-1.38) | 0.73 | 3.06 (1.34-7.48) | 0.31 | 0.80 (0.38-1.66) | 0.30 | 1.09 (0.59-2.00) | 0.79 |
| rs4315473 | C/T | 1.08 (0.78-1.49) | 0.27 | 0.59 (0.24-1.44) | 0.27 | 2.14 (0.93-5.16) | 0.73 | 0.83 (0.40-1.72) | 0.28 | 1.06 (0.81-1.38) | 0.68 |
| rs12465387 | G/A | 0.76 (0.43-1.29) | 0.08 | 1.08 (0.45-2.21) | 0.07 | 1.79 (0.53-6.14) | 0.10 | 0.57 (0.15-2.21) | 0.08 | 0.89 (0.59-1.33) | 0.56 |
| rs7608734 | G/A | 0.90 (0.62-1.28) | 0.20 | 1.19 (0.81-1.83) | 0.68 | 0.69 (0.30-1.53) | 0.28 | 1.47 (0.60-3.55) | 0.15 | 1.01 (0.79-1.36) | 0.95 |
| rs2701337 | G/A | 0.93 (0.68-1.26) | 0.40 | 1.50 (0.80-2.85) | 0.76 | 1.28 (0.69-2.44) | 0.58 | 1.74 (0.89-3.40) | 0.40 | 1.21 (0.88-1.68) | 0.25 |
| rs12636098 | C/T | 1.25 (0.89-1.73) | 0.24 | 0.89 (0.56-1.46) | 0.72 | 0.69 (0.29-1.55) | 0.25 | 0.45 (0.19-1.07) | 0.27 | 0.88 (0.59-1.31) | 0.53 |
| rs2129855 | T/C | 1.20 (0.90-1.60) | 0.45 | 0.81 (0.51-1.26) | 0.50 | 0.71 (0.33-1.49) | 0.47 | 1.36 (0.71-2.57) | 0.47 | 1.04 (0.79-1.36) | 0.80 |
| rs6599223 | C/T | 1.19 (0.82-1.72) | 0.17 | 1.05 (0.56-1.88) | 0.16 | 2.27 (0.90-6.19) | 0.83 | 1.61 (0.70-3.72) | 0.15 | 1.27 (0.96-1.68) | 0.10 |
| rs9859566 | A/G | 1.00 (0.72-1.38) | 0.27 | 1.04 (0.64-1.63) | 0.29 | 1.06 (0.49-2.26) | 0.31 | 1.84 (0.91-3.72) | 0.30 | 1.09 (0.86-1.38) | 0.48 |
| rs2399449 | T/C | 1.05 (0.78-1.41) | 0.37 | 1.04 (0.68-1.59) | 0.40 | 1.16 (0.56-2.43) | 0.66 | 1.59 (0.81-3.09) | 0.37 | 1.11 (0.89-1.38) | 0.36 |
| rs1806722 | T/C | 0.91 (0.64-1.28) | 0.24 | 1.02 (0.58-1.73) | 0.21 | 1.34 (0.58-3.14) | 0.20 | 1.01 (0.43-2.35) | 0.21 | 0.98 (0.75-1.27) | 0.87 |
| rs12695746 | G/T | 1.02 (0.67-1.52) | 0.16 | 1.15 (0.65-1.93) | 0.16 | 0.96 (0.39-2.26) | 0.82 | 0.52 (0.17-1.54) | 0.15 | 1.00 (0.74-1.19) | 0.98 |
| rs4683653 | T/C | 0.94 (0.69-1.28) | 0.32 | 1.01 (0.64-1.63) | 0.68 | 1.15 (0.56-2.42) | 0.34 | 0.55 (0.24-1.27) | 0.30 | 0.94 (0.74-1.19) | 0.60 |
| rs347684 | C/T | 1.10 (0.74-1.61) | 0.15 | 0.74 (0.35-1.41) | 0.14 | 1.12 (0.45-2.73) | 0.83 | 0.71 (0.25-1.98) | 0.15 | 0.98 (0.73-1.33) | 0.90 |
| rs347685 | A/C | 1.00 (0.73-1.38) | 0.29 | 0.78 (0.46-1.27) | 0.28 | 1.72 (0.85-3.57) | 0.73 | 0.58 (0.25-1.33) | 0.29 | 0.96 (0.75-1.22) | 0.75 |
| rs7683170 | C/A | 0.97 (0.73-1.30) | 0.32 | 1.23 (0.75-1.97) | 0.28 | 1.38 (0.64-3.02) | 0.28 | 1.18 (0.55-2.54) | 0.25 | 1.08 (0.86-1.35) | 0.53 |
| rs832540 | A/G | 1.04 (0.77-1.39) | 0.37 | 1.32 (0.84-2.11) | 0.60 | 1.32 (0.66-2.70) | 0.61 | 1.05 (0.52-2.16) | 0.36 | 1.13 (0.90-1.41) | 0.30 |
| rs832535 | T/C | 1.09 (0.81-1.47) | 0.37 | 0.85 (0.53-1.32) | 0.41 | 1.54 (0.78-3.13) | 0.60 | 1.45 (0.73-2.91) | 0.37 | 1.10 (0.88-1.37) | 0.42 |
| rs252894 | A/G | 1.09 (0.81-1.46) | 0.37 | 1.13 (0.73-1.79) | 0.59 | 1.54 (0.78-3.13) | 0.60 | 1.45 (0.73-2.91) | 0.37 | 1.17 (0.94-1.46) | 0.16 |
| rs245183 | T/G | 0.81 (0.59-1.10) | 0.29 | 1.27 (0.78-2.14) | 0.71 | 1.30 (0.57-3.03) | 0.72 | 1.20 (0.63-2.30) | 0.32 | 1.03 (0.76-1.39) | 0.86 |
| rs2681512 | A/G | 0.82 (0.60-1.11) | 0.30 | 0.88 (0.54-1.40) | 0.32 | 1.41 (0.69-2.99) | 0.69 | 1.14 (0.57-2.25) | 0.32 | 0.91 (0.73-1.15) | 0.44 |
| rs2250243 | T/C | 0.98 (0.72-1.33) | 0.28 | 1.11 (0.66-1.78) | 0.23 | 0.69 (0.29-1.56) | 0.24 | 0.60 (0.26-1.35) | 0.25 | 0.94 (0.74-1.19) | 0.62 |
| rs872424 | C/A | 0.98 (0.72-1.32) | 0.27 | 1.15 (0.70-1.85) | 0.23 | 0.65 (0.27-1.51) | 0.24 | 0.60 (0.27-1.33) | 0.25 | 0.94 (0.74-1.20) | 0.63 |
| rs1019179 | A/G | 0.66 (0.46-0.93) | 0.24 | 0.78 (0.43-1.33) | 0.23 | 1.32 (0.58-3.02) | 0.23 | 1.04 (0.53-2.04) | 0.28 | 0.80 (0.60-1.06) | 0.12 |
| rs10760840 | G/A | 1.14 (0.61-2.08) | 0.06 | 1.53 (0.68-3.14) | 0.07 | 0.45 (0.07-2.47) | 0.05 | 2.40 (0.72-8.02) | 0.05 | 1.31 (0.85-2.01) | 0.22 |
| rs10760846 | T/C | 1.38 (0.74-2.53) | 0.06 | 1.66 (0.73-3.39) | 0.07 | 0.50 (0.07-2.98) | 0.04 | 2.58 (0.76-8.71) | 0.04 | 1.50 (0.97-2.31) | 0.07 |
| rs10820084 | C/T | 1.38 (0.78-2.42) | 0.07 | 1.82 (0.90-3.44) | 0.08 | 1.35 (0.36-4.59) | 0.08 | 0.76 (0.16-3.72) | 0.06 | 1.46 (0.99-2.18) | 0.06 |
| rs13295426 | A/C | 1.23 (0.74-2.00) | 0.09 | 1.38 (0.63-2.69) | 0.08 | 0.39 (0.09-1.31) | 0.10 | 4.17 (1.66-10.49) | 0.08 | 1.39 (0.63-3.06) | 0.42 |
| rs12004778 | G/A | 1.01 (0.68-1.48) | 0.16 | 1.83 (1.04-3.12) | 0.15 | 2.00 (0.44-9.09) | 0.15 | 0.36 (0.13-0.98) | 0.21 | 1.07 (0.54-2.13) | 0.84 |
| rs2320 | T/G | 1.00 (0.75-1.32) | 0.43 | 1.14 (0.72-1.78) | 0.37 | 1.76 (0.86-3.80) | 0.40 | 0.79 (0.39-1.61) | 0.40 | 1.06 (0.85-1.31) | 0.62 |
| rs591510 | T/G | 1.01 (0.76-1.34) | 0.43 | 1.01 (0.64-1.58) | 0.37 | 1.93 (0.90-4.40) | 0.41 | 0.80 (0.39-1.63) | 0.40 | 1.04 (0.83-1.29) | 0.74 |
| rs633797 | G/A | 1.06 (0.80-1.41) | 0.43 | 1.02 (0.65-1.59) | 0.37 | 2.07 (0.91-5.03) | 0.41 | 0.80 (0.39-1.62) | 0.40 | 1.07 (0.86-1.33) | 0.54 |
| rs11605965 | C/T | 1.05 (0.67-1.62) | 0.11 | 1.23 (0.61-2.29) | 0.11 | 0.38 (0.10-1.25) | 0.11 | 0.64 (0.23-1.73) | 0.16 | 0.96 (0.69-1.34) | 0.82 |
| rs10783203 | G/A | 0.72 (0.53-0.99) | 0.33 | 0.97 (0.60-1.54) | 0.30 | 1.42 (0.68-3.00) | 0.69 | 1.04 (0.53-2.04) | 0.29 | 0.90 (0.68-1.19) | 0.47 |
| rs7971524 | G/T | 0.72 (0.53-0.99) | 0.33 | 0.97 (0.60-1.54) | 0.30 | 1.41 (0.67-2.96) | 0.68 | 1.02 (0.52-1.99) | 0.29 | 0.90 (0.68-1.18) | 0.44 |
| rs10784294 | G/A | 0.99 (0.74-1.32) | 0.39 | 1.23 (0.79-1.90) | 0.40 | 1.89 (0.94-4.00) | 0.57 | 1.36 (0.69-2.69) | 0.38 | 1.17 (0.91-1.49) | 0.22 |
| rs123165152 | A/C | 1.11 (0.60-1.99) | 0.06 | 2.19 (1.12-4.01) | 0.08 | 0.22 (0.02-1.18) | 0.05 |  |  | 1.18 (0.48-2.86) | 0.72 |
| rs1849384 | A/C | 0.99 (0.59-1.61) | 0.09 | 1.23 (0.64-2.17) | 0.12 | 0.81 (0.17-3.40) | 0.06 | 0.81 (0.16-4.01) | 0.06 | 1.04 (0.73-1.51) | 0.81 |
| rs10861973 | G/A | 0.89 (0.67-1.19) | 0.52 | 1.01 (0.66-1.55) | 0.48 | 0.98 (0.42-2.30) | 0.46 | 0.60 (0.28-1.25) | 0.46 | 0.89 (0.72-1.11) | 0.32 |
| rs12310859 | T/C | 1.13 (0.84-1.53) | 0.36 | 1.56 (0.99-2.45) | 0.38 | 0.69 (0.33-1.41) | 0.40 | 0.65 (0.30-1.38) | 0.38 | 1.04 (0.72-1.51) | 0.82 |
| rs2172326 | C/T | 1.10 (0.78-1.54) | 0.23 | 1.36 (0.82-2.20) | 0.24 | 0.97 (0.43-2.16) | 0.25 | 0.73 (0.33-1.64) | 0.27 | 1.10 (0.86-1.42) | 0.45 |
| rs7161311 | A/G | 1.10 (0.81-1.50) | 0.36 | 0.99 (0.64-1.53) | 0.37 | 1.95 (0.91-4.46) | 0.32 | 0.86 (0.45-1.63) | 0.36 | 1.09 (0.87-1.36) | 0.46 |
| rs12890808 | G/A | 0.94 (0.69-1.27) | 0.39 | 0.88 (0.56-1.34) | 0.38 | 1.12 (0.50-2.52) | 0.42 | 1.01 (0.49-2.07) | 0.38 | 0.94 (0.75-1.18) | 0.59 |
| rs4983025 | T/C | 0.91 (0.64-1.28) | 0.22 | 0.83 (0.48-1.38) | 0.23 | 2.32 (1.02-5.59) | 0.24 | 1.30 (0.63-2.72) | 0.26 | 1.07 (0.76-1.50) | 0.70 |
| rs12889256 | T/C | 0.95 (0.69-1.29) | 0.33 | 1.04 (0.66-1.61) | 0.33 | 1.50 (0.71-3.24) | 0.30 | 0.91 (0.46-1.80) | 0.35 | 1.01 (0.80-1.27) | 0.95 |
| rs11039 | T/G | 1.75 (1.13-2.72) | 0.12 | 1.02 (0.53-1.82) | 0.15 | 1.37 (0.55-3.44) | 0.18 | 1.37 (0.51-3.66) | 0.13 | 1.43 (1.03-1.99) | 0.03 |
| rs7342890 | T/C | 0.96 (0.66-1.38) | 0.19 | 1.25 (0.69-2.14) | 0.17 | 0.99 (0.46-2.11) | 0.75 | 1.08 (0.45-1.63) | 0.18 | 1.03 (0.79-1.36) | 0.81 |
| rs7220099 | A/G | 0.90 (0.62-1.29) | 0.19 | 1.30 (0.75-2.17) | 0.18 | 0.99 (0.45-2.13) | 0.75 | 1.04 (0.43-2.49) | 0.19 | 1.01 (0.77-1.32) | 0.94 |
| rs9896737 | G/A | 0.90 (0.62-1.29) | 0.19 | 1.34 (0.77-2.24) | 0.18 | 0.99 (0.45-2.13) | 0.75 | 1.04 (0.43-2.49) | 0.19 | 1.02 (0.78-1.33) | 0.90 |
| rs1791379 | C/A | 0.97 (0.69-1.36) | 0.26 | 0.96 (0.55-1.60) | 0.21 | 0.79 (0.26-2.33) | 0.74 | 1.05 (0.50-2.19) | 0.26 | 0.97 (0.74-1.25) | 0.79 |
| rs1862466 | G/A | 0.86 (0.64-1.17) | 0.48 | 0.91 (0.58-1.41) | 0.48 | 1.11 (0.54-2.29) | 0.49 | 0.87 (0.46-1.65) | 0.45 | 0.90 (0.72-1.12) | 0.34 |
| rs2833101 | G/A | 0.98 (0.74-1.31) | 0.49 | 0.69 (0.44-1.09) | 0.49 | 1.36 (0.67-2.81) | 0.54 | 0.61 (0.31-1.20) | 0.49 | 0.88 (0.67-1.14) | 0.32 |
| rs728174 | C/T | 1.00 (0.75-1.32) | 0.48 | 0.69 (0.43-1.09) | 0.46 | 1.36 (0.68-2.81) | 0.53 | 0.62 (0.32-1.22) | 0.49 | 0.88 (0.68-1.16) | 0.37 |
| rs2833117 | A/C | 1.07 (0.80-1.43) | 0.42 | 0.68 (0.42-1.07) | 0.41 | 0.70 (0.35-1.36) | 0.45 | 0.85 (0.44-1.63) | 0.42 | 0.87 (0.66-1.15) | 0.32 |
| rs4821872 | C/T | 1.26 (0.93-1.69) | 0.32 | 0.73 (0.43-1.18) | 0.30 | 0.91 (0.44-1.86) | 0.33 | 1.22 (0.57-2.62) | 0.31 | 0.91 (0.68-1.22) | 0.54 |

CI: confidence interval, E: effect allele, ER: emergency room, ESTATE: Effectiveness and Safety of Treatment with Asthma Therapy in children, OCS: oral corticosteroids; OR: odds ratio for the effect alleles, PACMAN: Pharmacogenetics of Asthma Medication in Children: Medication with Anti-inflammatory effects, PAGES: Paediatric Asthma Gene Environment Study, NA: no individuals in at least one comparison group, R: reference allele, SNP: single nucleotide polymorphism. All results were corrected for age, gender and principal components.

**Table S8. Results of associations of the selected meQTL with oral corticosteroid use in non-European childhood asthma cohorts**

|  |  | **SCSGES (n=139)** | | **SAGE**  **(n=562)** | | **GALA II**  **(n=976)** | | **Overall** |  |
| --- | --- | --- | --- | --- | --- | --- | --- | --- | --- |
| **SNP** | **Allele**  **(R/E)** | **OR (95%CI)** | **Allele frequency** | **OR (95% CI)** | **Allele frequency** | **OR (95% CI)** | **Allele frequency** | **OR (95% CI)** | **P-value** |
| rs241324 | A/G | 0.82 (0.40-1.69) | 0.56 | 0.93 (0.71-1.24) | 0.70 | 0.85 (0.68-1.08) | 0.77 | 0.90 (0.76-1.07) | 0.24 |
| rs6586361 | G/T | 2.12 (0.99-4.54) | 0.19 | 1.18 (0.88-1.59) | 0.67 | 1.09 (0.88-1.35) | 0.37 | 1.16 (0.98-1.37) | 0.09 |
| rs7604580 | G/A | 0.76 (0.31-1.87) | 0.17 | 1.13 (0.83-1.55) | 0.21 | 0.99 (0.78-1.26) | 0.23 | 1.03 (0.85-1.24) | 0.77 |
| rs4315473 | T/C | 1.70 (0.49-5.92) | 0.91 | 0.82 (0.63-1.07) | 0.48 | 1.00 (0.79-1.26) | 0.71 | 0.93 (0.77-1.12) | 0.44 |
| rs12465387 | G/A | 0.74 (0.31-1.78) | 0.17 | 0.84 (0.33-2.10) | 0.02 | 0.89 (0.62-1.28) | 0.09 | 0.86 (0.63-1.19) | 0.37 |
| rs7608734 | G/A | 0.34 (0.13-0.93) | 0.20 | 1.05 (0.80-1.38) | 0.55 | 0.86 (0.66-1.13) | 0.18 | 0.89 (0.68-1.16) | 0.38 |
| rs2701337 | A/G | 1.55 (0.71-3.36) | 0.76 | 1.22 (0.92-1.62) | 0.69 | 1.00 (0.82-1.22) | 0.59 | 1.10 (0.91-1.29) | 0.33 |
| rs12636098 | C/T | 0.93 (0.46-1.79) | 0.25 | 1.15 (0.85-1.57) | 0.26 | 1.10 (0.88-1.38) | 0.27 | 1.11 (0.93-1.32) | 0.25 |
| rs2129855 | T/C | 0.91 (0.46-1.79) | 0.36 | 0.94 (0.67-1.30) | 0.79 | 0.87 (0.71-1.06) | 0.54 | 0.89 (0.75-1.05) | 0.16 |
| rs6599223 | T/C | 0.60 (0.43-1.21) | 0.71 | 0.95 (0.67-1.34) | 0.81 | 0.99 (0.74-1.31) | 0.87 | 0.90 (0.74-1.10) | 0.32 |
| rs9859566 | A/G | 0.87 (0.33-2.30) | 0.15 | 1.12 (0.84-1.50) | 0.32 | 1.07 (0.86-1.33) | 0.29 | 1.08 (0.91-1.29) | 0.36 |
| rs2399449 | C/T | 0.67 (0.34-1.33) | 0.55 | 0.78 (0.55-1.09) | 0.20 | 1.03 (0.84-1.26) | 0.49 | 0.89 (0.69-1.15) | 0.37 |
| rs1806722 | T/C | 1.68 (0.68-4.15) | 0.13 | 0.86 (0.60-1.24) | 0.18 | 1.13 (0.88-1.44) | 0.22 | 1.06 (0.85-1.32) | 0.63 |
| rs12695746 | T/G | 1.51 (0.47-4.80) | 0.90 | 0.93 (0.53-1.64) | 0.94 | 1.08 (0.81-1.44) | 0.87 | 1.07 (0.83-1.37) | 0.61 |
| rs4683653 | T/C | 0.78 (0.36-1.69) | 0.26 | 1.17 (0.89-1.53) | 0.56 | 1.09 (0.88-1.35) | 0.33 | 1.10 (0.93-1.30) | 0.25 |
| rs347684 | T/C | 1.25 (0.51-3.08) | 0.82 | 0.64 (0.37-1.27) | 0.97 | 1.17 (0.85-1.61) | 0.90 | 1.03 (0.71-1.49) | 0.89 |
| rs347685 | C/A | 0.64 (0.64-2.88) | 0.29 | 0.79 (0.58-1.08) | 0.77 | 1.02 (0.80-1.29) | 0.78 | 0.95 (0.76-1.18) | 0.64 |
| rs7683170 | C/A | 0.74 (0.39-1.41) | 0.46 | 0.64 (0.37-1.10) | 0.09 | 0.79 (0.63-1.00) | 0.28 | 0.76 (0.62-0.94) | 0.01 |
| rs832540 | G/A | 0.54 (0.11-2.64) | 0.06 | 0.87 (0.63-1.19) | 0.25 | 0.85 (0.69-1.05) | 0.36 | 0.85 (0.72-1.18) | 0.07 |
| rs832535 | C/T | 0.54 (0.38-9.10) | 0.06 | 1.08 (0.82-1.42) | 0.38 | 0.83 (0.68-1.02) | 0.39 | 0.92 (0.72-1.18) | 0.51 |
| rs252894 | G/T | 0.54 (0.11-2.64) | 0.06 | 0.84 (0.59-1.18) | 0.21 | 0.84 (0.68-1.03) | 0.34 | 0.83 (0.70-0.99) | 0.04 |
| rs245183 | G/A | 2.14 (1.04-4.39) | 0.56 | 1.10 (0.84-1.44) | 0.63 | 0.96 (0.79-1.17) | 0.62 | 1.10 (0.85-1.41) | 0.46 |
| rs2681512 | G/T | 2.11 (1.05-4.21) | 0.52 | 1.07 (0.81-1.41) | 0.64 | 0.96 (0.79-1.17) | 0.60 | 1.10 (0.85-1.41) | 0.48 |
| rs2250243 | T/C | 0.88 (0.45-1.74) | 0.67 | 0.91 (0.63-1.31) | 0.16 | 0.88 (0.70-1.11) | 0.32 | 0.89 (0.74-1.07) | 0.21 |
| rs872424 | C/A | 1.06 (0.53-2.11) | 0.64 | 0.85 (0.60-1.22) | 0.18 | 0.86 (0.69-1.08) | 0.32 | 0.87 (0.73-1.05) | 0.15 |
| rs1019179 | A/G | 1.82 (0.25-1.19) | 0.25 | 1.11 (0.77-1.61) | 0.14 | 0.86 (0.69-1.08) | 0.28 | 1.04 (0.76-1.43) | 0.79 |
| rs10760840 | G/A | 1.67 (0.81-3.42) | 0.65 | 0.94 (0.62-1.41) | 0.13 | 0.88 (0.66-1.16) | 0.17 | 0.95 (0.76-1.18) | 0.64 |
| rs10760846 | T/C | 1.84 (0.96-3.52) | 0.56 | 0.88 (0.57-1.34) | 0.12 | 0.86 (0.65-1.15) | 0.16 | 1.02 (0.70-1.50) | 0.91 |
| rs10820084 | C/T | 1.65 (0.87-3.11) | 0.57 | 0.97 (0.57-1.67) | 0.07 | 0.97 (0.74-1.28) | 0.16 | 1.04 (0.83-1.31) | 0.73 |
| rs13295426 | A/C | 0.55 (0.12-2.61) | 0.06 | 0.83 (0.42-1.65) | 0.04 | 0.80 (0.52-1.24) | 0.05 | 0.79 (0.55-1.14) | 0.21 |
| rs12004778 | G/A | 1.80 (0.74-4.34) | 0.17 | 0.86 (0.60-1.22) | 0.19 | 0.85 (0.66-1.09) | 0.21 | 0.88 (0.73-1.08) | 0.22 |
| rs2320 | T/G | 1.01 (0.55-1.86) | 0.64 | 1.46 (1.06-2.00) | 0.21 | 0.78 (0.64-0.96) | 0.44 | 1.04 (0.69-1.57) | 0.86 |
| rs591510 | A/G | 1.00 (0.55-1.83) | 0.64 | 1.58 (1.09-2.30) | 0.15 | 0.78 (0.63-0.96) | 0.43 | 1.06 (0.67-1.66) | 0.81 |
| rs633797 | G/A | 0.99 (0.54-1.82) | 0.64 | 1.40 (0.97-2.00) | 0.17 | 0.78 (0.64-0.96) | 0.43 | 1.01 (0.69-1.48) | 0.97 |
| rs11605965 | C/T | 1.03 (0.40-2.67) | 0.16 | 1.73 (0.79-3.76) | 0.03 | 1.25 (0.85-1.84) | 0.08 | 1.30 (0.94-1.79) | 0.12 |
| rs10783203 | A/G | 0.83 (0.42-1.63) | 0.54 | 0.93 (0.70-1.25) | 0.70 | 0.97 (0.79-1.19) | 0.54 | 0.95 (0.81-1.12) | 0.53 |
| rs7971524 | T/C | 0.85 (0.43-1.67) | 0.54 | 0.97 (0.71-1.31) | 0.71 | 1.03 (0.84-1.26) | 0.55 | 1.00 (0.85-1.18) | 0.99 |
| rs10784294 | A/G | 1.61 (0.82-3.16) | 0.66 | 1.04 (0.81-1.34) | 0.54 | 1.01 (0.82-1.24) | 0.57 | 1.05 (0.89-1.22) | 0.58 |
| rs123165152 | A/C | NA | NA | NA | NA | NA | NA | NA | NA |
| rs1849384 | A/C | 0.90 (0.40-2.04) | 0.18 | 1.20 (0.87-1.66) | 0.75 | 0.94 (0.71-1.23) | 0.20 | 1.03 (0.84-1.28) | 0.77 |
| rs10861973 | G/A | NA | NA | 0.80 (0.60-1.07) | 0.66 | 1.08 (0.87-1.33) | 0.65 | 0.94 (0.71-1.26) | 0.69 |
| rs12310859 | T/C | 0.72 (0.35-1.51) | 0.27 | 1.08 (0.81-1.46) | 0.26 | 0.99 (0.79-1.24) | 0.25 | 1.00 (0.84-1.19) | 0.99 |
| rs2172326 | C/T | 1.13 (0.11-2.47) | 0.78 | 1.15 (0.74-1.77) | 0.11 | 1.10 (0.84-1.46) | 0.16 | 1.10 (0.89-1.37) | 0.42 |
| rs7161311 | T/G | 4.16 (1.86-9.30) | 0.37 | 1.23 (0.93-1.62) | 0.53 | 1.16 (0.93-1.43) | 0.33 | 1.65 (0.81-3.36) | 0.17 |
| rs12890808 | G/A | 2.18 (1.10-4.34) | 0.31 | 1.04 (0.79-1.36) | 0.58 | 1.05 (0.84-1.30) | 0.31 | 1.09 (0.92-1.28) | 0.31 |
| rs4983025 | T/C | NA | NA | 0.93 (0.69-1.27) | 0.29 | 1.23 (0.94-1.61) | 0.16 | 1.08 (0.83-1.42) | 0.57 |
| rs12889256 | T/C | 1.27 (0.60-2.72) | 0.21 | 0.78 (0.59-1.02) | 0.43 | 0.98 (0.79-1.22) | 0.29 | 0.91 (0.74-1.12) | 0.37 |
| rs11039 | T/G | 1.02 (0.39-2.64) | 0.12 | 0.73 (0.37-1.43) | 0.05 | 1.14 (0.83-1.56) | 0.11 | 1.05 (0.79-1.38) | 0.74 |
| rs7342890 | C/T | 0.96 (0.31-2.06) | 0.92 | 1.04 (0.76-1.41) | 0.75 | 0.90 (0.73-1.12) | 0.69 | 0.94 (0.80-1.12) | 0.51 |
| rs7220099 | G/A | 0.79 (0.30-2.06) | 0.91 | 0.96 (0.73-1.25) | 0.43 | 1.07 (0.87-1.32) | 0.63 | 1.02 (0.86-1.20) | 0.84 |
| rs9896737 | A/G | 0.93 (0.32-2.68) | 0.92 | 1.05 (0.80-1.38) | 0.53 | 1.02 (0.82-1.26) | 0.65 | 1.03 (0.87-1.21) | 0.76 |
| rs1791379 | A/G | 1.81 (0.82-4.00) | 0.76 | 1.38 (0.74-2.58) | 0.95 | 0.96 (0.73-1.26) | 0.83 | 1.17 (0.81-1.70) | 0.40 |
| rs1862466 | A/G | 0.84 (0.45-1.55) | 0.63 | 0.74 (0.54-0.99) | 0.30 | 1.04 (0.85-1.28) | 0.42 | 0.89 (0.68-1.15) | 0.38 |
| rs2833101 | G/A | 1.58 (0.75-3.37) | 0.71 | 0.94 (0.70-1.27) | 0.68 | 0.98 (0.78-1.22) | 0.64 | 0.99 (0.83-1.18) | 0.91 |
| rs728174 | C/T | 1.55 (0.80-2.99) | 0.42 | 1.02 (0.76-1.37) | 0.32 | 0.92 (0.74-1.15) | 0.57 | 0.99 (0.83-1.17) | 0.88 |
| rs2833117 | A/C | 1.71 (0.86-3.40) | 0.44 | 0.96 (0.73-1.26) | 0.45 | 0.94 (0.76-1.16) | 0.57 | 0.98 (0.83-1.15) | 0.79 |
| rs4821872 | C/T | 1.91 (0.82-4.43) | 0.17 | 0.96 (0.67-1.36) | 0.16 | 1.11 (0.89-1.38) | 0.29 | 1.09 (0.91-1.31) | 0.33 |

CI: confidence interval, ER: emergency room, GALA II: Genes-environments & Admixture in Latino Americans study, NA: no individuals in at least one comparison group, OCS: oral corticosteroids, OR: odds ratio, SAGE: Study of African Americans, Asthma, Genes and Environments, SCSGES: Singapore Cross Sectional Genetic Epidemiology Study, SNP: single nucleotide polymorphism. All results were corrected for age, gender and principal components.

| **Table S9. Pharmacogenomic analysis focusing on genotype-by-ICS treatment effect on 3 years of FEV1 changes Lung Health Study (LHS)-2 (COPD)** | | | | | | | | | | |  |  |
| --- | --- | --- | --- | --- | --- | --- | --- | --- | --- | --- | --- | --- |
| **SNP** | **CHR** | **BP** | **Rsq** | **REF** | **ALT** | **MAF** | **Genotyped** | | **se** | **P** | **beta** | **OR** |
| rs12316152 | 12 | 85719558 | 0.99941 | A | C | 0.04889 | Imputed |  | 0.018944 | 3.18E-03 | 5.61E-02 | 1.05766 |
| rs10784294 | 12 | 62822067 | 0.99965 | A | G | 0.38871 | Genotyped | | 0.008438 | 2.89E-02 | 1.85E-02 | 1.018637 |
| rs2320 | 10 | 5930512 | 0.99996 | T | G | 0.38701 | Genotyped | | 0.008332 | 5.26E-02 | -1.62E-02 | 0.983955 |
| rs11605965 | 11 | 62154161 | 0.99986 | C | T | 0.12141 | Genotyped | | 0.013121 | 5.73E-02 | 2.50E-02 | 1.025295 |
| rs633797 | 10 | 5972365 | 0.99993 | G | A | 0.38821 | Genotyped | | 0.008315 | 7.32E-02 | -1.49E-02 | 0.985195 |
| rs591510 | 10 | 5941953 | 0.997 | A | G | 0.39053 | Imputed |  | 0.008328 | 8.26E-02 | -1.45E-02 | 0.985631 |
| rs4691233 | 4 | 1.57E+08 | 0.99989 | A | G | 0.34824 | Genotyped | | 0.008492 | 1.02E-01 | -1.39E-02 | 0.986186 |
| rs252894 | 5 | 56224339 | 0.99945 | G | T | 0.36086 | Imputed |  | 0.008522 | 1.12E-01 | 1.35E-02 | 1.013637 |
| rs832535 | 5 | 56213334 | 0.9992 | C | T | 0.35716 | Imputed |  | 0.008609 | 1.16E-01 | 1.35E-02 | 1.013632 |
| rs7683170 | 4 | 1.57E+08 | 0.98149 | C | A | 0.29093 | Imputed |  | 0.008864 | 1.26E-01 | -1.36E-02 | 0.986526 |
| rs832540 | 5 | 56199202 | 0.99989 | G | A | 0.35542 | Genotyped | | 0.008585 | 1.30E-01 | 1.30E-02 | 1.013105 |
| rs1862466 | 19 | 21164399 | 0.99956 | A | G | 0.45319 | Genotyped | | 0.008199 | 1.44E-01 | 1.20E-02 | 1.012071 |
| rs2513081 | 11 | 62092267 | 0.99998 | A | G | 0.17467 | Genotyped | | 0.010758 | 1.51E-01 | 1.54E-02 | 1.015569 |
| rs2725236 | 4 | 88919106 | 0.99988 | G | A | 0.47479 | Genotyped | | 0.00826 | 1.59E-01 | -1.16E-02 | 0.988432 |
| rs12890808 | 14 | 25285800 | 0.99999 | G | A | 0.36373 | Genotyped | | 0.008583 | 1.82E-01 | -1.15E-02 | 0.988598 |
| rs7220099 | 17 | 36784639 | 0.99978 | G | A | 0.17844 | Genotyped | | 0.010539 | 2.35E-01 | 1.25E-02 | 1.012612 |
| rs7342890 | 17 | 36777379 | 0.99767 | C | T | 0.1789 | Imputed |  | 0.010556 | 2.38E-01 | 1.25E-02 | 1.012544 |
| rs10917023 | 1 | 21925603 | 0.99987 | A | G | 0.069 | Genotyped | | 0.016514 | 2.55E-01 | 1.88E-02 | 1.019006 |
| rs9896737 | 17 | 36790736 | 0.97839 | A | G | 0.17857 | Imputed |  | 0.010638 | 2.86E-01 | 1.14E-02 | 1.011433 |
| rs4821872 | 22 | 39600651 | 0.99611 | C | T | 0.32432 | Imputed |  | 0.008679 | 3.27E-01 | -8.51E-03 | 0.991529 |
| rs2528584 | 2 | 1.6E+08 | 0.99948 | C | T | 0.20663 | Imputed |  | 0.010005 | 3.38E-01 | 9.59E-03 | 1.00964 |
| rs6586361 | 1 | 2.35E+08 | 0.99826 | G | T | 0.48544 | Genotyped | | 0.008162 | 3.74E-01 | -7.25E-03 | 0.992772 |
| rs2833117 | 21 | 32200972 | 0.9999 | A | C | 0.4371 | Genotyped | | 0.008234 | 3.87E-01 | -7.13E-03 | 0.992897 |
| rs7608734 | 2 | 2.37E+08 | 0.98189 | G | A | 0.18334 | Imputed |  | 0.010525 | 4.01E-01 | 8.85E-03 | 1.008884 |
| rs728174 | 21 | 32186269 | 0.99665 | C | T | 0.49123 | Imputed |  | 0.008139 | 4.39E-01 | -6.30E-03 | 0.993718 |
| rs2833101 | 21 | 32185088 | 0.99963 | G | A | 0.49518 | Imputed |  | 0.008182 | 4.47E-01 | -6.23E-03 | 0.993791 |
| rs6599223 | 3 | 38650460 | 0.99993 | T | C | 0.15517 | Genotyped | | 0.01179 | 4.69E-01 | -8.54E-03 | 0.991498 |
| rs7161311 | 14 | 25275554 | 0.99962 | T | G | 0.35608 | Genotyped | | 0.008656 | 4.71E-01 | -6.25E-03 | 0.99377 |
| rs347685 | 3 | 1.42E+08 | 0.99914 | C | A | 0.26368 | Imputed |  | 0.009657 | 5.67E-01 | -5.53E-03 | 0.994489 |
| rs1791379 | 18 | 3611838 | 0.99131 | A | G | 0.26684 | Genotyped | | 0.009232 | 5.70E-01 | 5.25E-03 | 1.005266 |
| rs2681512 | 5 | 1.27E+08 | 0.99929 | G | T | 0.33033 | Imputed |  | 0.008784 | 5.74E-01 | -4.94E-03 | 0.99507 |
| rs10861973 | 12 | 1.09E+08 | 0.98089 | G | A | 0.49774 | Imputed |  | 0.008537 | 5.77E-01 | -4.77E-03 | 0.995245 |
| rs10819242 | 9 | 1.3E+08 | 0.96474 | C | T | 0.09618 | Imputed |  | 0.014535 | 5.79E-01 | 8.08E-03 | 1.008108 |
| rs2701337 | 2 | 2.38E+08 | 0.99492 | A | G | 0.38557 | Imputed |  | 0.008677 | 5.87E-01 | 4.72E-03 | 1.004728 |
| rs1806722 | 3 | 1.28E+08 | 0.98125 | T | C | 0.22222 | Imputed |  | 0.00974 | 6.50E-01 | 4.43E-03 | 1.004435 |
| rs10783203 | 12 | 47929308 | 0.99458 | A | G | 0.29569 | Imputed |  | 0.008946 | 6.55E-01 | 3.99E-03 | 1.004002 |
| rs2172326 | 12 | 1.3E+08 | 0.99958 | C | T | 0.23974 | Genotyped | | 0.009726 | 6.60E-01 | 4.28E-03 | 1.004293 |
| rs7604580 | 2 | 1.59E+08 | 0.99999 | G | A | 0.29985 | Genotyped | | 0.008948 | 6.67E-01 | -3.86E-03 | 0.99615 |
| rs854334 | 14 | 25329433 | 0.99982 | C | A | 0.30978 | Genotyped | | 0.008725 | 6.73E-01 | 3.68E-03 | 1.003689 |
| rs7971524 | 12 | 47935320 | 0.99963 | T | C | 0.29525 | Genotyped | | 0.008931 | 6.76E-01 | 3.73E-03 | 1.003735 |
| rs11607961 | 11 | 1.23E+08 | 0.99949 | T | C | 0.20913 | Imputed |  | 0.010281 | 6.85E-01 | 4.18E-03 | 1.004186 |
| rs11607961 | 11 | 1.23E+08 | 0.99949 | T | C | 0.20913 | Imputed |  | 0.010281 | 6.85E-01 | 4.18E-03 | 1.004186 |
| rs347684 | 3 | 1.42E+08 | 0.99942 | T | C | 0.13268 | Imputed |  | 0.012506 | 6.98E-01 | 4.86E-03 | 1.004872 |
| rs12004778 | 9 | 1.26E+08 | 0.97939 | G | A | 0.18958 | Imputed |  | 0.010212 | 7.04E-01 | 3.88E-03 | 1.003887 |
| rs7619506 | 3 | 1.42E+08 | 0.99014 | A | G | 0.15444 | Imputed |  | 0.011512 | 7.11E-01 | 4.27E-03 | 1.004274 |
| rs2250243 | 7 | 6690240 | 0.99963 | T | C | 0.24824 | Genotyped | | 0.009768 | 7.23E-01 | 3.47E-03 | 1.003471 |
| rs872424 | 7 | 6698905 | 0.9993 | C | A | 0.24843 | Genotyped | | 0.009786 | 7.23E-01 | 3.47E-03 | 1.003472 |
| rs4683653 | 3 | 1.42E+08 | 0.98538 | T | C | 0.30501 | Imputed |  | 0.009232 | 7.24E-01 | 3.26E-03 | 1.003264 |
| rs241324 | 1 | 2.29E+08 | 0.99854 | A | G | 0.16863 | Genotyped | | 0.011003 | 7.49E-01 | -3.52E-03 | 0.996482 |
| rs241324 | 1 | 2.29E+08 | 0.99854 | A | G | 0.16863 | Genotyped | | 0.011003 | 7.49E-01 | -3.52E-03 | 0.996482 |
| rs245183 | 5 | 1.27E+08 | 0.994 | G | A | 0.3068 | Imputed |  | 0.008926 | 7.52E-01 | 2.82E-03 | 1.002822 |
| rs12695746 | 3 | 1.42E+08 | 0.99999 | T | G | 0.16126 | Genotyped | | 0.011153 | 7.60E-01 | 3.41E-03 | 1.003418 |
| rs12636098 | 3 | 11974696 | 0.99189 | C | T | 0.27119 | Imputed |  | 0.009621 | 7.69E-01 | -2.83E-03 | 0.997178 |
| rs12636098 | 3 | 11974696 | 0.99189 | C | T | 0.27119 | Imputed |  | 0.009621 | 7.69E-01 | -2.83E-03 | 0.997178 |
| rs1019179 | 7 | 38218976 | 0.99981 | A | G | 0.23433 | Genotyped | | 0.009756 | 7.94E-01 | 2.54E-03 | 1.002548 |
| rs796051 | 12 | 89740864 | 0.98045 | T | C | 0.20592 | Imputed |  | 0.010325 | 8.20E-01 | -2.35E-03 | 0.997651 |
| rs11039 | 14 | 52897619 | 0.96578 | T | G | 0.13444 | Imputed |  | 0.012057 | 8.93E-01 | -1.62E-03 | 0.99838 |
| rs12310859 | 12 | 1.3E+08 | 0.99997 | T | C | 0.36324 | Genotyped | | 0.008758 | 9.01E-01 | 1.09E-03 | 1.001093 |
| rs12310859 | 12 | 1.3E+08 | 0.99997 | T | C | 0.36324 | Genotyped | | 0.008758 | 9.01E-01 | 1.09E-03 | 1.001093 |
| rs2129855 | 3 | 26768818 | 0.96811 | T | C | 0.46933 | Imputed |  | 0.0082 | 9.49E-01 | 5.22E-04 | 1.000522 |
| rs4649058 | 1 | 25375468 | 0.9998 | G | A | 0.41242 | Genotyped | | 0.008302 | 9.76E-01 | 2.53E-04 | 1.000253 |
| rs2399449 | 3 | 1.12E+08 | 0.99982 | C | T | 0.35728 | Genotyped | | 0.008754 | 9.83E-01 | 1.83E-04 | 1.000183 |
| rs9859566 | 3 | 1.12E+08 | 0.99849 | A | G | 0.28911 | Imputed |  | 0.009122 | 9.95E-01 | 5.89E-05 | 1.000059 |
| rs13295426 | 9 | 1.17E+08 | 0.99962 | A | C | 0.08976 | Genotyped | | 0.013863 | 9.99E-01 | 1.65E-05 | 1.000017 |

OR: odds ratio, All results were corrected for age, gender and principal components. MAF: Minor allele frequency

**
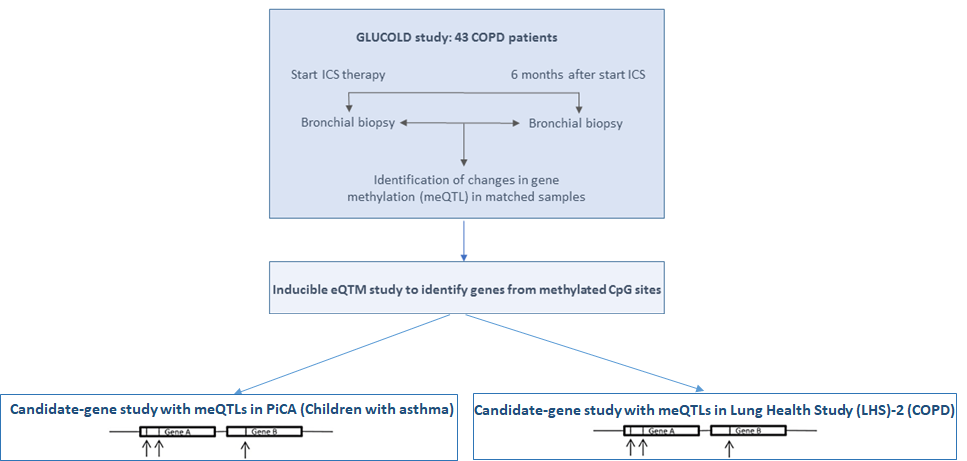
**

**Figure S1. Flow diagram of the study procedures.** COPD: Groningen and Leiden Universities study of Corticosteroids in Obstructive Lung Disease, eQTM: expression Quantitative Trait Methylation, SNPs: single nucleotide polymorphisms, GLUCOLD: Groningen and Leiden Universities study of Corticosteroids in Obstructive Lung Disease, ICS: Inhaled corticosteroids, meQTL: methylation Quantitative Trait Loci. , PiCA: Pharmacogenetics in Childhood Asthma consortium, SNPs: single nucleotide polymorphisms.

**Supplementary information 1. Methylation methods**

Methylation

Genomic DNA was bisulfite converted using the EZ DNA Methylation Gold Kit (Zymo Research) and used for microarray-based DNA methylation analysis, performed at GenomeScan (GenomeScan B.V., Leiden, The Netherlands) on the HumanMethylation850 BeadChip (Illumina, Inc., San Diego, CA, U.S.A). This array interrogates over 850,000 CpG sites representing about 99% of the RefSeq genes. The bisulfite-converted DNA was processed and hybridised to the HumanMethylation850 BeadChip (Illumina, Inc.), according to the manufacturer's instructions. The BeadChip images were scanned on the iScan system and the data quality was assessed using the R script MethylAid (M. van Iterson, 2014) using default analysis settings.

**Supplementary information 2. Cohorts included in the meta-analysis of the ICS candidate-gene study**

**BREATHE (n=113)**

The BREATHE cohort includes children and young adults (age 3-22 years) with asthma based on the diagnosis of the physician. They were recruited in primary and secondary care units in Scotland, United Kingdom. Clinical history, demographic, and anthropometric information was obtained from all participants between 2004 and 2006[9-11]. Genotyping was performed using Illumina Infinium CoreExome-24 BeadChip (Illumina).

**GALA II (n=996)**

The Genes-environments & Admixture in Latino Americans study (GALA II) included Latino patients (age 8-21 years) with a physician diagnosis of asthma with active symptoms and asthma medication use during the last 2 years. Information about genetics, environment, exacerbations and medication use was obtained. Genotyping was performed with the Axiom® LAT1 array (Affymetrix Inc.) and Quality Control (QC) is described elsewhere[12].

**PAGES (n=442)**

The Paediatric Asthma Gene Environment Study (PAGES) included children (age: 2-16 years) with a physician’s diagnosis of asthma in 15 hospitals in Scotland between 2008 and 2011. Genetic, data describing exhaled NO and spirometry, and information about asthma symptoms, diet, medication, exacerbations, allergies and quality of life were obtained[13]. Genotyping was performed using the Axiom^TM^ Precision Medicine Research Array (Affymetrix Inc.) by Centro Nacional de Genotipado in Spain (CeGen; www.cegen.org).

**PACMAN (n=704)**

The Pharmacogenetics of Asthma Medication in Children: Medication with Anti-inflammatory effects (PACMAN) cohort is an observational cohort with children (age 4-12 years) with self-reported (regular) use of asthma medication. The children were recruited via Dutch community pharmacies. Asthma symptoms, exacerbations and medication use were collected during 12 months between 2009 and 2012[14]. LGC Genomics (UK) performed the genotyping with the Illumina Infinium CoreExome-24 BeadChip (Illumina). Visual inspection of the genotypes was performed as QC even as exclusion of patients with missing genotype call rate > 0.50. SNPs were excluded when the missing genotype rate > 0.01, Hardy-Weinberg equilibrium p-value < 0.001 or MAF < 0.01.

**SAGE (n=567)**

The Study of African Americans, Asthma, Genes and Environments (SAGE) included African American patients (age 8-21 years) with a physician diagnosis of asthma with active symptoms and asthma medication use. Information about genetics, environment, exacerbations and medication use was obtained[15]. The genotyping data was retrieved using the same methods as GALA II[15].

**Singapore Cross Sectional Genetic Epidemiology Study (n=139)**

The Singapore Cross Sectional Genetic Epidemiology Study (SCSGES) included atopic patients with a Singapore Chinese ethnicity. The study population is of Chinese ethnicity, living in Singapore and all subjects are not related to each other. Genetic and medication data and information about allergies was obtained. Genotyping was performed using the Illumina HumanHap 550 k BeadChip version 3 (Illumina).

**ESTATe (n=101)**

The Effectiveness and Safety of Treatment with Asthma Therapy in children (ESTATE) is a case-control study including children and young adults aged four to nineteen years with a physician diagnosis of asthma recruited from primary care practices in the Netherlands. A cohort with children with asthma aged five years and older was selected within this case-control study and they were invited to participate via their general practitioner. After their written consent, their answers to research questionnaires and saliva samples for DNA extraction were collected. Genotyping was carried out with the Illumina Infinium CoreExome-24 BeadChip (Illumina). Exclusion was performed in SNPs and samples with a call rate < 99% or Hardy-Weinberg p-value < 0.001 as QC. Samples with excess autosomal heterozygosity were also excluded.

**SLOVENIA (n=175)**

The SLOVENIA cohort included children with asthma diagnosed according to American Thoracic Society (ATS) criteria and aged between five and eighteen years old. During inclusion, they had newly detected mild or moderate persistent asthma. Data describing their asthma symptoms, exacerbations and clinical outcomes were obtained during the physician visit over the preceding twelve months. Genotyping was performed using high resolution melting analysis (HRMA) on LightCycler 480 instrument (Roche, Germany) using LC480 HRM Master Mix (Roche, Germany), under the conditions of initial denaturation at 95°C in 10 min, followed by 45 cycles of 95°C for 10S, 60°C for 15S and 72°C for 10S, a HRM step of 95°C for 1 min, 40°C for 1 min and 60-90°C at 0.02°C/s. Afterwards genotypes were determined using gene scanning analysis software.
